# Aromatic Dendrimers Bearing 2,4,6-Triphenyl-1,3,5-triazine Cores and Their Photocatalytic Performance

**DOI:** 10.1021/acs.joc.1c00039

**Published:** 2021-04-22

**Authors:** Jakub S. Cyniak, Artur Kasprzak

**Affiliations:** Faculty of Chemistry, Warsaw University of Technology, Noakowskiego Str. 3, 00-664 Warsaw, Poland

## Abstract

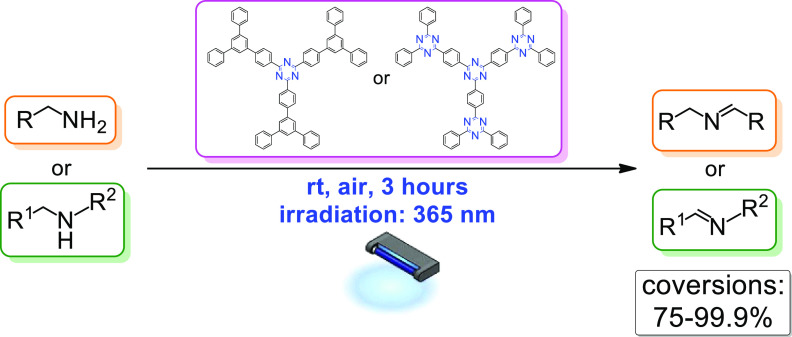

The synthesis of two novel aromatic dendrimers structurally derived from 1,3,5-tri[1,3-diphenyl(phenyl-5-yl)phenyl-4′-yl]benzene and bearing 2,4,6-triphenyl-1,3,5-triazine cores is reported. The obtained dendrimers were used for the OLEDs construction, as well as in the role of innovative photocatalysts for the very efficient and selective oxidation of various benzylamines to respective *N*-benzylidene benzylamines under mild conditions.

Aromatic dendrimers represent an important class of highly ordered and monodisperse molecules, the properties and functions of which can be tuned by the selection of different cores and dendrons.^1,2^ Over the past 20 years, low-generation aromatic dendrimers^3^ bearing 1,2,3-triazine skeletons have been intensively explored, because of their encouraging properties and functions as well as many prospective applications. It is noteworthy that aromatic dendrimers bearing a 2,4,6-triphenyl-1,3,5-triazine skeleton as the core molecule feature improved thermal stability and beneficial photophysical properties associated with improved electron-transfer processes.^4−6^ Thus, many examples of 2,4,6-triphenyl-1,3,5-triazine-based aromatic dendrimers were reported in the literature, with fluorene,^4,7−9^ carbazole,^10−12^ pyrene,^13^ phenoxazine,^14^ bithiophene,^15^ oxadiazole,^16^ or long-chain alkoxyl groups^17^ acting as a dendron motif. The above-mentioned molecules were found to exhibit beneficial light-emission properties, e.g., toward the construction of organic-light emitting diodes^4,11−15^ or liquid crystalline materials.^16,17^

We envisioned that from the structural viewpoint, 1,3,5-tri[1,3-diphenyl(phenyl-5-yl)phenyl-4′-yl]benzene (**1**; Figure 1) can be termed as a very original structure for the works dealing with the preparation of aromatic dendrimers consisting of 2,4,6-triphenyl-1,3,5-triazine cores. The first reports on the synthesis of **1** were published in 1973–2003.^18−22^ Since then many interesting examples of the respective low-generation aromatic dendrimers showing beneficial light-emission-related features were reported, as discussed above. However, we have noted that the synthesis of (2,4,6-triphenyl-1,3,5-triazine)-containing dendrimers **D1** and **D2** (Figure 1), which can be claimed as directly structurally derived from **1**, were not reported to date. We believe that filling this gap will provide valuable insights into the chemistry and photophysical properties of aromatic dendrimers consisting of 2,4,6-triphenyl-1,3,5-triazine skeletons. Thus, the aim of this work was to synthesize two symmetric aromatic dendrimers bearing one (**D1**) or four (**D2**) 1,3,5-triazine cores, as well as the evaluation of their photophysical properties toward organic light-emitting diod (OLED) device construction. Furthermore, for the first time we present the fully innovative photocatalytic performance for such a class of aromatic dendrimers. Retrosynthetic analysis for **D1** and **D2** is presented in Figure 2a.

**Figure 1:**
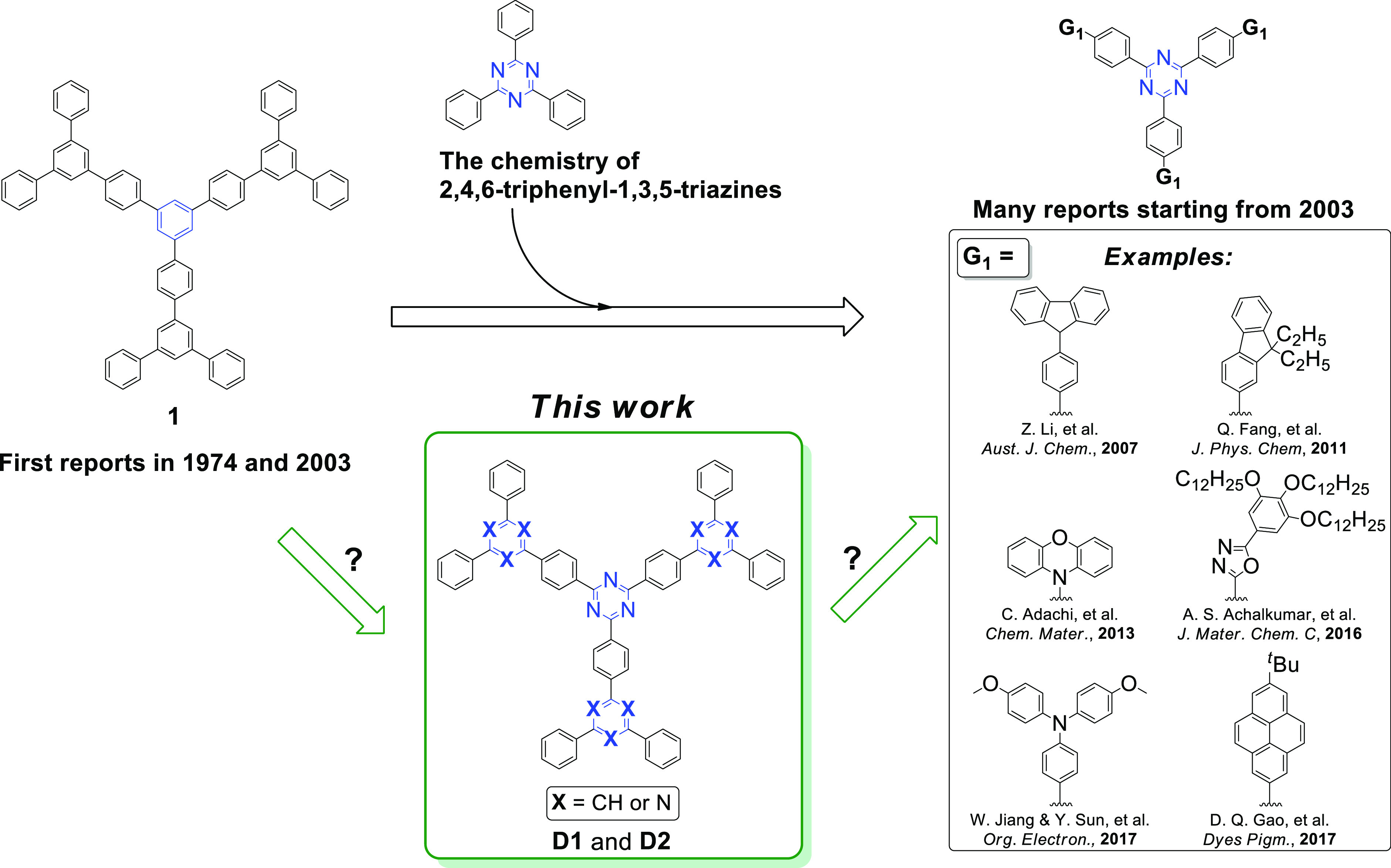
Graphical representation of the aim of this work.

**Figure 2:**
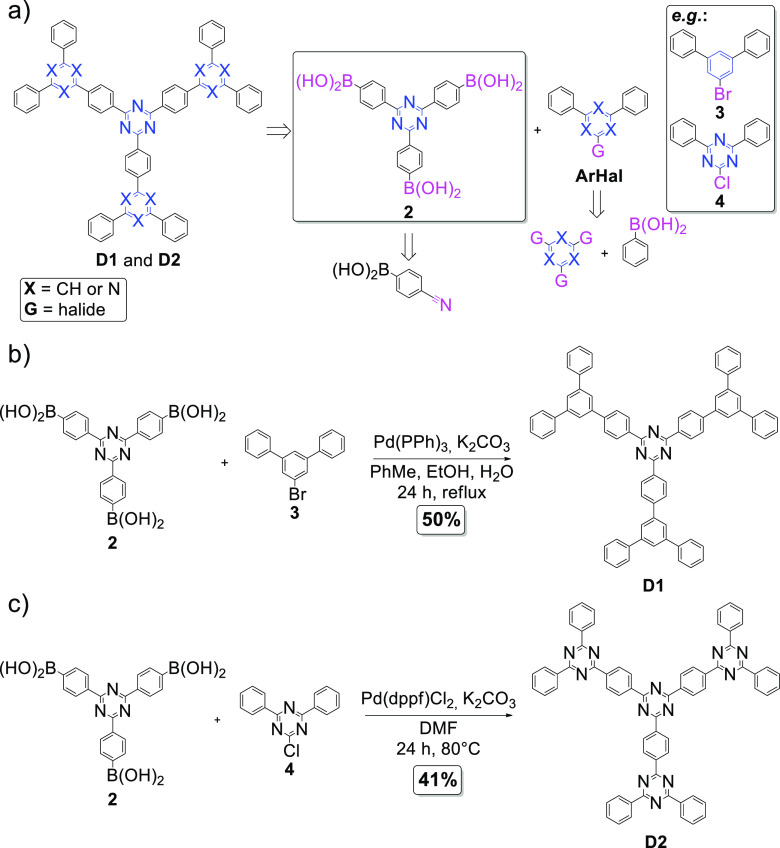
(a) Retrosynthetic analysis for **D1** and **D2**. (b) Synthesis path to obtain **D1**. (c) Synthesis path to obtain **D2**.

We envisioned that **D1** and **D2** could be synthesized via the Suzuki–Miyaura cross-coupling reactions starting from ((1,3,5-triazine-2,4,6-triyl)tris(benzene-4,1-diyl))triboronic acid (**2**) and the respective aryl halides (**ArHal**; Figure 2a). We hypothesized that this methodology can be especially attractive because starting materials are commercially available or can be easily synthesized. As for example, **2** was easily obtained via the acid-catalyzed trimerization of 4-cyanophenylboronic acid,^23^ while 1-bromo-3,5-diphenylbenzene (**3**) was fabricated via Suzuki–Miyaura cross-coupling reaction starting from 1,3,5-tribromobenzene.^24^

To our delight, compound **D1** was successfully synthesized from **2** and 1-bromo-3,5-diphenylbenzene (**3**) (Figure 2b). Optimization experiments (Table S1) revealed that the highest yield (50%) was archived with the use of tetrakis(triphenylphosphine)palladium(0) as a catalyst and K_2_CO_3_ as a base in toluene/ethanol/water solvent system under reflux conditions. The formation of pure **D1** was confirmed spectroscopically (^1^H NMR, ^1^H–^1^H COSY NMR, ^13^C NMR, FT-IR) and using the high-resolution mass spectrometry (HRMS) together with elemental analysis (see Supporting Information for the full compound characterization data). It is noteworthy that ^1^H NMR analysis revealed the presence of six sets of signals for **D1**, which means that the **D1** is indeed symmetric.

The successful synthesis of **D2**, which comprises four 1,3,5-triazine skeletons, was achieved with the treatment of **2** with the excess of 2-chloro-4,6-diphenyl-1,3,5-triazine (**4**) (Figure 2c). The reaction has been investigated by changing various reaction parameters, such as a type of a palladium catalyst, reaction temperature, and the type of a solvent (Table S2). Under optimized reaction conditions that employed [1,1′-bis(diphenylphosphino)ferrocene]dichloropalladium(II) as a catalyst, K_2_CO_3_ as a base in DMF at 80 °C, **D2** was synthesized with 41% yield. Dendrimer **D2** was subjected to NMR analyses (^1^H NMR, ^1^H–^1^H COSY NMR, ^13^C NMR, FT-IR) and HRMS, while the purity of the sample has been confirmed with elemental analysis (see Supporting Information for the full compound characterization data). Three sets of signals in ^1^H NMR revealed that **D2** is symmetric.

The profiles of UV–vis spectra of **D1** and **D2** (Figure S25) were similar. Both **D1** and **D2** exhibited two strong absorption maxima (λ_max_) located at ca. 250 nm and ca. 325 nm. Slight red shift (ca. 5 nm) and higher molar absorption coefficient value for λ_max_ = 323 nm than for λ_max_ = 254 nm was found for **D2**. The observed absorption maxima were ascribed to the π–π* transitions originating from the presence of benzene and 1,3,5-triazine moieties.^9,16,17^ Significant changes in the emission intensity for **D1** and **D2** were observed (Figure S25). Both **D1** and **D2** exhibited higher emission intensity values for excitation wavelength (λ_ex_) of 315 nm in comparison to λ_ex_ = 250 nm. **D2** featured ca. 3-fold higher emission intensity value in comparison to **D1**. Emission maximum (λ_em_) for **D2** was also red-shifted (ca. 40 nm) in comparison to **D1**. These changes were ascribed to the higher content of 1,3,5-triazine skeletons in **D2** (four units) in comparison to **D1** (one unit) and the expansion of a π-conjugation system.^5,17,25^ Only slight changes between the emission spectra measured in other selected solvents were found (Figures S10–S11). Emission quantum yields (Φ_F_) (λ_ex_ = 315 nm) estimated by the relative method^26^ were 0.32 and 0.78, for **D1** and **D2**, respectively.

Considering these satisfactory emission properties, preliminary OLED application trials with **D1** and **D2** were performed.^13^ The experimental details of devices construction are listed in Section S6. Device consisting of **D2** exhibited better performance than the respective device composed of **D1** (Table S4). For the **D2**-based device, the maximum external quantum efficiency equaled to 2.82%. Additionally, this device exhibited turn-on voltage at the level of 3.4 V and featured the maximum luminance of 5515 cd·m^–2^ at 8.9 V together with higher current density values than the respective **D1**-consisting device (Figure S26; Table S4). These values suggest that the as-fabricated OLED devices are characterized by the satisfactory or comparable values in comparison with similar aromatic dendrimers for OLED applications reported in the literature (Table S4).^11−15^ Thus, **D1** and **D2** might be considered as new examples of aromatic dendrimers exhibiting good light emission properties.

Encouraged by the efficient light-emission properties exhibited by **D1** and **D2**, we also investigated a fully innovative application of these aromatic dendrimers as the photocatalysts in the oxidation of benzylamine to *N*-benzylidene benzylamine. Finding new ways of selective oxidative coupling of amines yielding the respective imines, which serve as important building blocks in organic synthesis and industry, is of a highest importance. The reported catalytic systems commonly include toxic metal complexes together with harsh reaction conditions, such as high temperature or high pressure.^27−29^ Thus, there is a continuous interest in the design of new, sustainable, and efficient approaches for aerobic oxidation of amines to respective imines.^30−38^ In the recent years there has been a great progress in designing novel photocatalysts dedicated to such oxidation processes. These studies include, e.g., the use of novel BODIPY,^39,40^ phenoxazine,^41,42^ or salicylic acid^43^ derivatives as effective photocatalysts, also for the aerobic oxidation processes.^39,40^

We hypothesized that newly synthesized **D1** or **D2** might work as an efficient catalyst in this photocatalytic process. First, there are literature reports on the use of 1,3,5-triazine-containing polymers or materials in photocatalytic oxidation of primary amines.^37−44^ We supposed that **D2** might exhibit better catalytic performance than **D1** because of above-discussed enhanced light-emission properties arising from the higher content of pyridinic nitrogen atoms.^38^ Second, HOMO–LUMO band gaps estimated for **D1** and **D2** by the Tauc plot method^45^ (3.5–3.6 eV; see Section S3) suggest that oxidation as well as electron transfer processes in the studied reaction should be feasible. The literature points that the mechanism^37,46^ would include the following key transformations: (i) generation of an excited state of **D1**/**D2** (D1*/D2*) that reduces O_2_ to its active species: superoxide radical (O_2_^•–^) and singlet oxygen (^1^O_2_), (ii) oxidation of benzylamine to cationic intermediate by D1*/D2* that regenerates a catalyst (for the literature plausible reaction mechanism, see Section S3). To enable such reaction pathway, the LUMO level of a photocatalyst should be higher than the respective value for oxygen (−3.8 eV), while the HOMO level should be higher than HOMO of benzylamine (−5.9 eV). Relatively high values of HOMO–LUMO band gaps for **D1** and **D2** shall meet these criteria, which was further evidenced with the cyclic voltammetry (CV) experiments (Figures S17–S19).

The performance of **D1** and **D2** as photocatalysts in the selective oxidation of benzylamine (**5**) to *N*-benzylidene benzylamine (**6**) is summarized in Table S3. The reactions were carried out on air at room temperature under irradiation with UV-LED as a light source (wavelength of light of 365 nm). **D1** and **D2** exhibited the photocatalytic performance. **D2** provided more satisfactory reaction results in comparison to **D1** (Table S3, entries 1–2). In the preliminary trial with 0.5 mol % of the photocatalyst **D2**, the conversion of 78% was observed in 1.5 h. Increasing the reaction time (3 h) and the amount of the catalyst (2.0 mol %) provided practically quantitative conversion of the reactants together with the quantitative isolated yield (Table S3, entry 6). Additionally, further purification of the reaction mixture was not required under optimized conditions. Isolation of pure *N*-benzylidene benzylamine (**6**) was confirmed with ^1^H NMR and elemental analysis (data in the Experimental Section). It should be stressed that no conversion was found in the absence of the photocatalyst or without light irradiation (Table S3, entries 7–8). Additionally, significantly lower conversions (34–47%) were found when the photocatalytic reactions were performed in the presence of benzoquinone (Table S3, entry 9) or 5,5-dimethyl-1-pyrroline *N*-oxide (DMPO; Table S3, entry 10), the superoxide radical (O_2_^•–^) scavengers. These results support the role of O_2_^•–^ in the studied oxidation coupling that was further evidenced by the measurement of electron paramagnetic resonance (EPR) spectra (the characteristic signal originating from the presence of DMPO-O_2_^•–^ species was clearly observed; Figure S20).^32^ To support the role of ^1^O_2_ in the studied photocatalytic process, the reaction was performed in the presence of 1,4-diazabicyclo[2.2.2]octane (DABCO; Table S3, Entry 11). The reaction was significantly suppressed by the DABCO (conversion 20%), which supports that ^1^O_2_ was involved in the photocatalytic process. We also found that **D2** is characterized by the ca. 2.4-fold longer fluorescence lifetime (2.9 μs) in comparison to **D1** (1.2 μs; see Figures S21–S22), which, we anticipate, influenced on the better photocatalytic performance of **D2** in comparison to **D1**.

The results of our photocatalytic experiments under mild conditions suggest that newly synthesized photocatalyst **D2** has a higher or comparable rate in comparison with other literature protocols employing a photocatalytic approach (Table 1). Importantly, our methodology is metal-free and the process occurs under mild conditions. **D2** is also characterized by better catalytic performance (higher or comparable conversions in shorter times) in comparison with the respective triazine-containing materials (Table 1, entries 4, 5). Notably, beneficial reaction conversions (≥99%) with very low amounts of a photocatalyst were observed in several cases, only, while 2 mol % of **D2** is enough to quantitatively convert the substrate in the product. Our homogeneous photocatalyst **D2** can be also easily separated from the reaction mixture and recycled up to ten reaction cycles without any loss of its remarkable catalytic activity (see Section S3). While such recyclability has been observed for the reported heterogeneous photocatalysts, we found recyclability perspective for the new efficient homogeneous photocatalyst **D2** as very important factor from the practical viewpoint that stands for the novelty of our protocol. Additionally, with our approach it is possible to quantitatively isolate pure *N*-benzylidene benzylamine (**6**) in a chromatography-free way. Ultimately, **D1** and **D2** were also characterized by the good thermal stability, as elucidated by the thermogravimetric analyses (Figures S23–S24).

**Table 1:** Comparison between the Photocatalytic Performance in the Synthesis of *N*-Benzylidene Benzylamine (**6**) for **D2** and Other Selected Photocatalysts

entry	photocatalyst	amount of the catalyst	light source	conversion (%)	time (h)	temperature	isolated yield (%)	TON^a^	TOF^b^ [h^–1^]	ref.
**1**	**D2**	**2 mol %**	**UV-LED**	**99.8 ± 0.2**	**3.0**	**rt**	**quantitative**	**49.9**	**16.6**	**this work**
2	1%Pt@TiO2–500	25 mg^c^^,^^d^	Xe lamp	99.8	16	rt	not given	N/A^e^	N/A^e^	(30)
3	BiOCl nanosheets	15 mol %	visible light	87.6	10	rt	84.9	5.84	0.6	(31)
4	mpg-C_3_N_4_	25 mg^c^^,^^d^	visible light	99.0	3.5	80 °C	90.0	N/A^e^	N/A^e^	(37)
5	Triazine-containing conjugated porous polymers	3 mg^c^^,^^d^	LED	>99.0	5.0	rt	not given	N/A^e^	N/A^e^	(38)
6	Phenol-TiO_2_ complex	8 mg^c^^,^^d^	LED	95	1.6	rt	87	N/A^e^	N/A^e^	(50)
7	TFPT-BMTH	5 mol %	LED	99	24	rt	not given	19.8	0.8	(32)
8	Py-BSZ-COF	12.5 mg^a^^,^^b^	LED	99	12	rt	not given	N/A^e^	N/A^e^	(33)
9	Zn-PDI	1 mol %	LED	74	4	40 °C	not given	74	18.5	(51)
10	pTCP-2P	0.5 mol %	visible light	98	6	rt	not given	196	32.6	(34)
11	BiOBr-OV	25 mg^c^^,^^d^	Xe lamp	96	12	rt	not given	N/A^e^	N/A^e^	(35)
12	SC-HM	10 mg^c^^,^^d^	Xe lamp	99	4	rt	not given	N/A^e^	N/A^e^	(36)
13	B-BO-1,3,5	3 mg^c^^,^^d^	LED	99	24	rt	not given	N/A^e^	N/A^e^	(52)
14	Tx-CMP	1 mol %	Xe lamp	96	4	60 °C	not given	96	24	(53)
15	NH_2_-MOL 125 Ti	25 mg^c^^,^^d^	Xe lamp	73	12	rt	not given	N/A^e^	N/A^e^	(54)
16	CF-HCP	12.5 mg^c^^,^^d^	LED	91	6	rt	80.6	N/A^e^	N/A^e^	(55)

a Turnover number.^57^

b Turnover frequency.^57^

c Insoluble material (heterogeneous catalyst), mol % amount of the catalyst was not provided.

d Per 0.5 mmol of benzylamine.

e TON and TOF values were not determined^57^ (no information about mol % of the catalyst or the number of active sites was provided).

The applicability of our methodology has been further elucidated by using different amines for this reaction (Figure 3). We found that benzylamines bearing electron-withdrawing groups or electron-donating groups can undergo this photocatalytic oxidative coupling. High conversions^47^ (>75%) toward the formation of respective *N*-benzylidene benzylamines were observed. Conversions for the benzylamines with electron-donating groups (ca. 84–99%) were higher than for the respective benzylamines bearing electron-withdrawing functionalities (ca. 75–93%). Conversions for ortho- or meta-substituted benzylamines (ca. 75–84%) were lower than for the para isomers (ca. 93–99%), which was attributed to steric effects.^37^ With our approach, heterocyclic benzylamines (**16**–**19**) can also be efficiently synthesized (>88%), which demonstrates the applicability of the designed photocatalytic protocol. Ultimately, selected secondary amines (**20**–**23**) were also included in our studies. It was found that using **D2** as the photocatalyst in such process provides very satisfactory reaction results (>80%).

**Figure 3:**
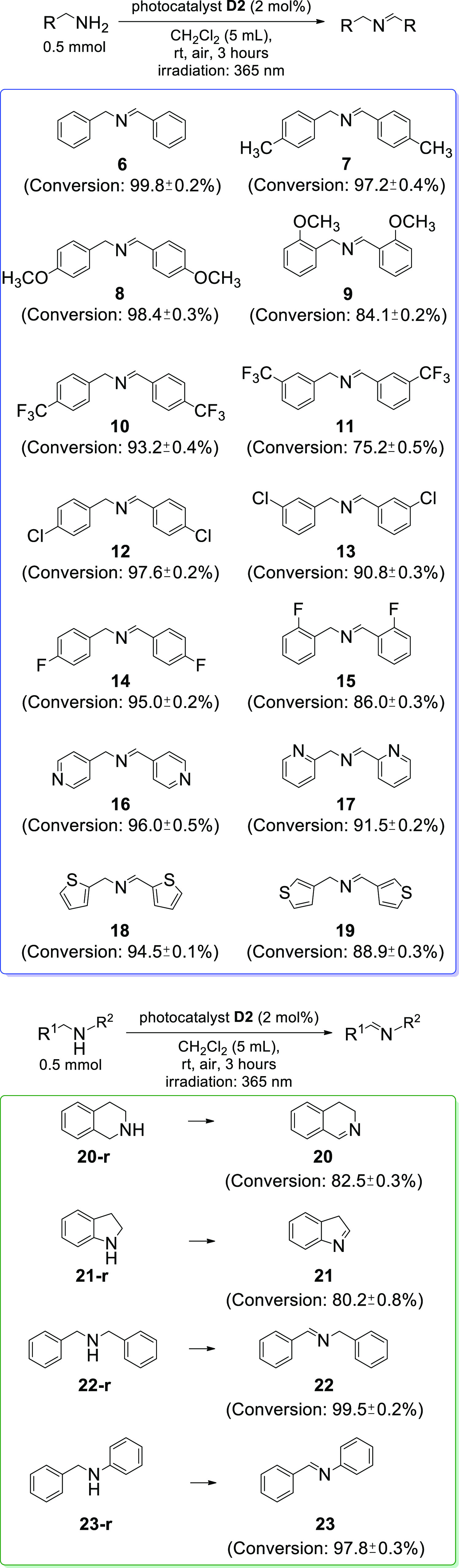
Scope of the designed photocatalytic oxidation. Conversions were estimated with ^1^H NMR. Reaction conditions: amine (0.5 mmol), **D2** (2 mol %), CH_2_Cl_2_ (5 mL), room temperature, air, 3 h, UV-LED irradiation (365 nm).

In conclusion, we reported the synthesis of two new aromatic dendrimers **D1** and **D2** bearing one or four 1,3,5-triazine skeletons, respectively. The Suzuki–Miyaura cross-coupling reactions starting from ((1,3,5-triazine-2,4,6-triyl)tris(benzene-4,1-diyl))triboronic acid (**2**) provided the target products in 41–50% yields. **D1** and **D2** showed beneficial light-emission properties, as evidenced by the spectroscopic analyses and preliminary OLED application tests. We demonstrated that **D1** and **D2** can act as innovative photocatalysts in the selective oxidative coupling of benzylamines. Quantitative yield in the selective synthesis of *N*-benzylidene benzylamine was achieved in short reaction time (3 h) under mild conditions with the use of only 2.0 mol % of the photocatalyst **D2**. The designed photocatalyst is also reusable, and various benzylamines can be subjected to this process. This work fills the gap in the chemistry of aromatic dendrimers directly structurally derived from 1,3,5-tri[1,3-diphenyl(phenyl-5-yl)phenyl-4′-yl]benzene and bearing 2,4,6-triphenyl-1,3,5-triazine cores. It also sheds a very new light on applications of this class of aromatic molecules in photocatalysis. Our future works will include the preparation of functionalized aromatic dendrimers **D1** and **D2** and their applications in light-emitting materials’ sciences.

## Experimental Section

### Materials and Methods

Chemical reagents and solvents were commercially purchased and purified according to the standard methods, if necessary. The NMR experiments were carried out using a Varian VNMRS 500 MHz spectrometer (^1^H NMR at 500 MHz or ^13^C NMR at 125 MHz) equipped with a multinuclear z-gradient inverse probe head. The spectra were recorded at 25 °C and standard 5 mm NMR tubes were used. ^1^H and ^13^C chemical shifts (δ) were reported in parts per million (ppm) relative to the solvent signal, i.e., CDCl_3_, δ_H_ (residual CHCl_3_) 7.26 ppm, δ_C_ 77.2 ppm. NMR spectra were analyzed with the MestReNova v12.0 software (Mestrelab Research S.L.). UV–vis measurements were performed with a SPECORD S 600 Spectrophotometer with the spectral resolution of 1 nm. PL measurements were performed with a Hitachi F-4500 fluorescence spectrophotometer with the spectral resolution of 1 nm. For the UV–vis and PL measurements, the wavelengths for the absorption or emission maxima λ_max_ were reported in nm. Fourier-transform infrared (FT-IR) spectra were recorded in the attenuated total reflectance (ATR) mode with the Thermo Nicolet Avatar 370 spectrometer with spectral resolution of 2 cm^–1^ (80 scans). The wavenumbers for the absorption bands ν were reported in cm^–1^. ESI-HRMS (TOF) measurements were performed with a Q-Exactive Thermo Scientific spectrometer. Elemental analyses were performed using CHNS Elementar Vario EL III apparatus. Each elemental composition was reported as an average of two analyses. TLC analysis was performed using Merck Silica gel 60 F254 plates. Oil baths with a temperature control unit were used for the reactions that required heating. Electron paramagnetic resonance (EPR) spectra were measured with JEOL JES–FA200 EPR spectrometer. Photocatalytic reactions were performed with a Spectroline E-Series UV-LED lamp, Spectronics, Corp. (2 × 3.0 W; λ = 365 nm; lamp power 22 000 μW·cm^–1^; UV intensity >4500 μW·cm^–2^; borosilicate glass test tubes (diameter: 1.0 cm, maximum volume: 8 mL) were used in the photocatalytic tests; the irradiation distance: 3 cm; no filters were used). Cyclic voltammetry (CV) measurements were performed with a ALS/CH Electrochemical Analyzer, conditions: CH_2_Cl_2_, compound **D1**/**D2**: 0.15 mM, 0.1 M tetra-*n*-butylammonium fluoride (TBAF), scan rate; 0.10 V·s^–1^. Thermogravimetric analyses (TGA) were performed with a Netzsch STA449C thermobalance under argon atmosphere with a heating rate of 10 °C·min^–1^.

1-Bromo-3,5-diphenylbenzene (**3**)^48^ and ((1,3,5-triazine-2,4,6-triyl)tris(benzene-4,1-diyl))triboronic acid (**2**)^49^ were prepared following the literature procedures, while **4** (purity ≥98%) was purchased from AmBeed, USA.

### Synthesis of **D1**

((1,3,5-Triazine-2,4,6-triyl)tris(benzene-4,1-diyl))triboronic acid (**2**; 8.8 mg, 0.02 mmol), 1-bromo-3,5-diphenylbenzene (**3**; 21.6 mg, 0.07 mmol), Pd(PPh_3_)_4_ (6.9 mg, 0.006 mmol) and K_2_CO_3_ (33.2 mg, 0,24 mmol) were refluxed in PhMe:EtOH:H_2_O (2.1 mL: 0.7 mL: 0.7 mL) for 24 h. Reaction mixture was extracted with CH_2_Cl_2_ (3 × 15 mL). The organic layers were combined, dried with MgSO_4_, filtered, and the solvent was removed in a vacuum. The resultant residue was purified by column chromatography (SiO_2_; 25% CHCl_3_/hex) to give **D1** (8.9 mg, 50% yield) as the white solid.

^1^H NMR (CDCl_3_, 500 MHz, ppm), δ_H_ 8.95–8.89 (m, 6H), 7.95–7.90 (m, 12H), 7.86–7.85 (m, 3H), 7.76–7.74 (m, 12H), 7.54–7.50 (m, 12H), 7.44–7.42 (m, 6H); ^13^C{^1^H} NMR (CDCl_3_, 125 MHz, ppm), δ_C_ 171.6, 145.3, 145.3, 142.8, 141.7, 141.2, 135.7, 129.8, 129.1, 127.8, 127.6, 126.1, 125.4; FT-IR (ATR), *v* 2915, 2846, 1569, 1508, 1423, 1366, 1012, 806, 745, 686 cm^–1^; HRMS (ESI-TOF) *m*/*z* [M + H]^+^ Calcd. for C_75_H_52_N_3_ 994.4161. Found: 994.4164. Anal. Calcd for C_75_H_51_N_3_: C, 90.60; H, 5.15; N, 4.23. Found: C, 90.59; H, 5.16; N, 4.25; *R*_*f*_ (25% CHCl_3_/hex) = 0.23.

### Synthesis of **D2**

A solution of ((1,3,5-Triazine-2,4,6-triyl)tris(benzene-4,1-diyl))triboronic acid (**2**; 26.7 mg, 0.06 mmol), 2-chloro-4,6-diphenyl-1,3,5-triazine (**4**; 96.4 mg, 0.36 mmol), Pd(dppf)Cl_2_ (4.4 mg, 0.006 mmol) and K_2_CO_3_ (100.0 mg, 0.72 mmol) in dry DMF (6.0 mL) was heated at 80 °C for 24 h under argon atmosphere. Reaction mixture was extracted with CH_2_Cl_2_ (3 × 15 mL). The organic layers were combined, dried with MgSO_4_, filtered, and the solvent was removed in a vacuum. The resultant residue was purified by column chromatography (SiO_2_; 50% CHCl_3_/hex) to give **D2** (24.4 mg, 41% yield) as the white solid.

^1^H NMR (CDCl_3_, 500 MHz, ppm), δ_H_ 8.66–8.63 (m, 15H), 7.61–7.51 (m, 26H); ^13^C{^1^H} NMR (CDCl_3_, 125 MHz, ppm), δ_C_ 173.7, 171.7, 135.9, 123.8, 129.2, 128.8, 128.7; FT-IR (ATR), *v* 2912, 2844, 1524, 1483, 1363, 1234, 1074, 837, 740, 683 cm^–1^; HRMS (ESI-TOF) *m*/*z* [M + H]^+^ Calcd. for C_66_H_43_N_12_ 1003.1201. Found: 1003.1205. Anal. Calcd for C_66_H_42_N_12_: C, 79.02; H, 4.22; N, 16.76. Found: C, 79.04; H, 4.23; N, 16.73; *R*_*f*_ (50% CHCl_3_/hex) = 0.56.

### Photocatalytic Synthesis of *N*-Benzylidene Benzylamines from Benzylamines

#### Optimized Protocol

A round-bottom reaction flask was charged with the photocatalyst (**D1**/**D2**; optimized conditions: **D2**, 2 mol %) and CH_2_Cl_2_ (5 mL) was added. Benzylamine or its derivative (0.5 mol) was added. The reaction mixture was stirred on air at room temperature under irradiation with the wavelength of light of 365 nm for a given time (optimized conditions: 3 h). Solvent was then evaporated, and the mixture was diluted with CH_3_CN. This procedure was performed to separate the photocatalyst (**D1**/**D2**; not soluble in CH_3_CN) from the crude product. Solid photocatalyst has been filtrated and dried under a high vacuum to recover **D1**/**D2**, while the supernatant (containing the product) was evaporated, dried under a high vacuum, and subjected to ^1^H NMR to determine the conversion of benzylamines (for the method of determination of the conversion, see Subsection S3.2). Each experiment was performed three times to check the reproducibility of the results.

Benzylamines **6**–**23**, which are known compounds for which literature ^1^H NMR data matched the ^1^H NMR data in our catalytic experiments, were obtained following the above-listed optimized protocol.

Under optimized conditions photocatalyst **D2** was quantitatively recovered from the reaction mixture and then reused in next reaction cycles. For the results of these reusability studies, see Figure S12.

Comparison between the photocatalytic performance in the synthesis of *N*-benzylidene benzylamine(**6**) for **D2** and other photocatalysts reported in the literature is presented in Table 1.

### Data for the Pure *N*-Benzylidene Benzylamine (**6**) Isolated under Optimized Conditions

^1^H NMR (CDCl_3_, 500 MHz, ppm), δ_H_ 8,41 (m, 1H), 7.81–7.78 (m, 2H), 7.44–7.40 (m, 3H), 7.36–7.35 (m, 4H), 7.30–7.25 (m, 1H), 4.84 (s, 2H). NMR data are consistent with the literature.^56^^1^H NMR spectrum of **6** is presented in Figure S13. Anal. Calcd for C_14_H_13_N: C, 86.12; H, 6.71; N, 7.17. Found: C, 86.15; H, 6.70; N, 7.15.

## Supporting Information Available

Tables dealing with the optimization experiments, compounds characterization data, photocatalytic experiments data, EPR spectrum, CV data, TGA data (PDF)
