# Correction to Aromatic Dendrimers Bearing 2,4,6-Triphenyl-1,3,5-triazine Cores and Their Photocatalytic Performance

**DOI:** 10.1021/acs.joc.1c01548

**Published:** 2021-07-20

**Authors:** Jakub S. Cyniak, Artur Kasprzak

It has come to our attention that the NMR spectra provided for compound **D2** were accidentally incorrect. The Supporting Information has been updated to present correct NMR spectra (Figures S4–S7). The reported NMR signals for compound **D2** listed in the experimental section (page 6, main article) *should be replaced by*: “^1^H NMR (CDCl_3_, 500 MHz, ppm), δ_H_ 8.79–8.76 (m, 12H), 8.00 (s, 12H), 7.62–7.55 (m, 18H); ^13^C{^1^H} NMR (CDCl_3_, 125 MHz, ppm), δ_C_ 173.3, 172.1, 171.5, 136.1, 134.3, 133.4, 132.4, 129.3, 128.9, 128.7, 128.5; (...)”. We would also like to present ESI-HRMS spectra of **D1** and **D2** that were not provided in the original Supporting Information (Figures S27–S28 were added to the Supporting Information, section S7).

Also, in Table S2 (Supporting Information), the titles of second and third column *should be replaced by*: second column - “**2** (mg; *mmol*; equiv)”, third column - “**4** (mg; *mmol*; equiv)”.

The authors would like to thank Prof. Sergiusz Luliński (Warsaw University of Technology, Poland) for fruitful discussions.

## Supporting Information Available

Corrected file containing tables dealing with the optimization experiments, compounds characterization data, photocatalytic experiments data, EPR spectrum, CV data, TGA data (PDF)

