# Supplementary material for: Correction to Aromatic Dendrimers Bearing 2,4,6-Triphenyl-1,3,5-triazine Cores and Their Photocatalytic Performance
Source: J Org Chem. 2021 Jul 20;86(15):10940. doi: 10.1021/acs.joc.1c01548 (PMC8504788; doi:10.1021/acs.joc.1c01548)
Supplement: Supplementary file 1 — jo1c01548_si_001.pdf [file jo1c01548_si_001.pdf]

Aromatic dendrimers bearing 2,4,6-triphenyl-1,3,5-triazine cores and their photocatalytic performance

Supporting Information

Jakub S. Cyniak and Artur Kasprzak*

Faculty of Chemistry, Warsaw University of Technology, Noakowskiego Str. 3, 00-664 Warsaw, Poland

* corresponding author e-mail: akasprzak@ch.pw.edu.pl

Table of CONTENTS

S1. Optimization experiments toward the synthesis of **D1** and **D2** S2

S2. Compounds characterization data S5

S3. Photocatalytic studies S14

S3.1 Photocatalytic synthesis of *N*-benzylidene benzylamines from benzylamines S14

S3.2 Determination of conversion of benzylamine based on ^{1}H NMR spectra...S16

S3.3 ^{1}H NMR spectrum of the isolated *N*-benzylidene benzylamine (**6**) under optimized conditions S17

S34.4 Determination of HOMO-LUMO band gaps for **D1** and **D2**......................S18

S3.5 Plausible literature reaction mechanism S18

S4. TGA curves for **D1** and **D2**...S23

S5. UV-Vis spectra S24

S6. OLED application trials with **D1** and **D2**...S25

S7. ESI-HRMS spectra of **D1** and **D2** S28

S8. References..S29

S1. Optimization experiments toward the synthesis of D1 and D2

The data for optimization experiments are summarized in Table S1 and Table S2 located below.

Table S1. Optimization experiments for the synthesis of **D1**.

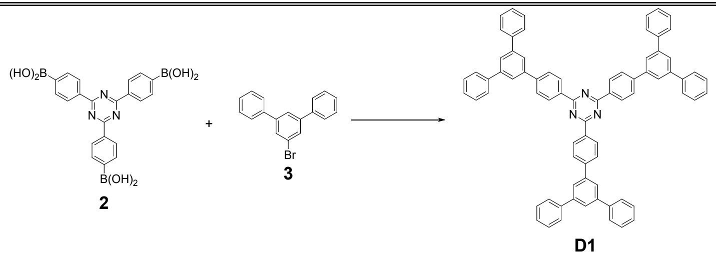

**Entry**	**2** (mg; *mmol*; equiv)	**3** (mg; *mmol*; equiv)	**solvent** (ml)	**catalyst** (mg; *mmol*; equiv)	**base** (mg; *mmol*; equiv)	**Reaction time** (hours)	**temp.** (°C)	**D1 yield**[a] (mg; %)
1	8.8; *0.02*; 1.0	21.6; *14.28*; 3.5	PhMe (2.1); H_2O (0.7); EtOH (0.7)	$Pd(PPh_3)_4$ 3.5; 0.003; 0.15	Na_2CO_3 21.2; 0.2; 10	24	reflux	4.6; 23.0
2	8.8; *0.02*; 1.0	21.6; *0.07*; 3.5	DMF (3.5)	$Pd(dppf)Cl_2$ 2.2; 0.003; 0.15	CH_3COOK 19.6; 0.2; 10	24	reflux	3.4; 17.0
3	8.8; *0.02*; 1.0	21.6; *0.07*; 3.5	PhMe (2.1); H_2O (0.7); EtOH (0.7)	$Pd(PPh_3)_4$ 3.5; 0.003; 0.15	Na_2CO_3 21.2; 0.2; 10	48	reflux	4.1; 21.0
4	8.8; *0.02*; 1.0	21.6; *0.07*; 3.5	PhMe (2); EtOH (2)	$Pd(dppf)Cl_2$ 2.2; 0.003; 0.15	K_2CO_3 33.2; 0.24; 12	24	reflux	4.8; 24.0
5	8.8; *0.02*; 1.0	21.6; *0.07*; 3.5	PhMe (2); EtOH (2)	XPhos Pd G2 4.7; 0.005; 0.3 XPhos 5.7; 0.01; 0.6	K_2CO_3 33.2; 0.24; 12	24	reflux	7.7; 39.0
6	8.8; *0.02*; 1.0	21.6; *0.07*; 3.5	PhMe (2.1); H_2O (0.7); EtOH (0.7)	$Pd(PPh_3)_4$ 6.9; 0.006; 0.3	K_2CO_3 33,2; 0.24; 12	24	reflux	8.9; **50.0**

[a] Isolated yield.

Table S2. Optimization experiments for the synthesis of **D2**.

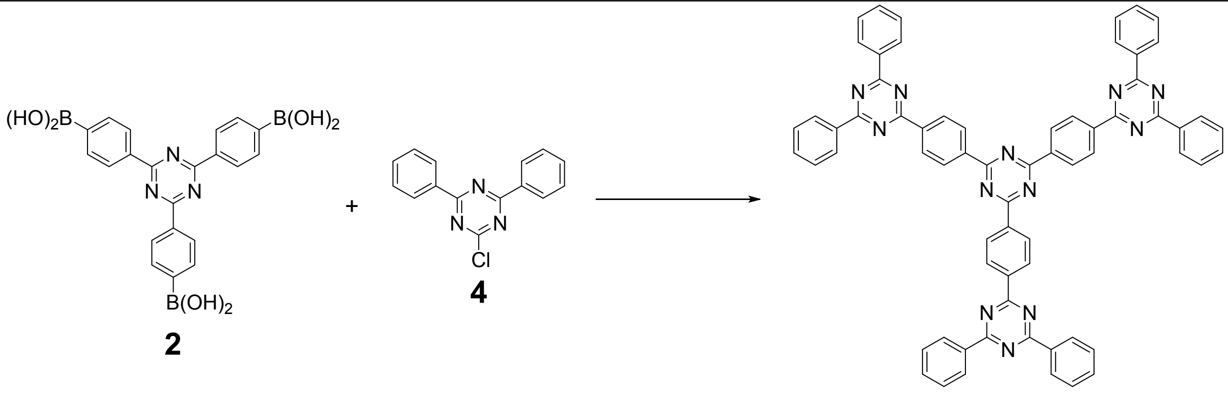

2 + **4** → **D2**

Entry	**2**<br>(mg;<br>*mmol*;<br>equiv)	**4**<br>(mg;<br>*mmol*;<br>equiv)	**solvent**<br>(ml)	**catalyst**<br>(mg; *mmol*;<br>equiv)	**base**<br>(mg; *mmol*;<br>equiv)	**Reaction time**<br>(hours)	**temp.**<br>(°C)	**D2 yield**[a]<br>(mg; %)
1	26.7;<br>0.06;<br>1.0	57.0;<br>0.21;<br>3.5	PhMe<br>(6.5);<br>H_2O (3);<br>THF (5)	$Pd(PPh_3)_4$<br>7.0; 0.006;<br>0.1	K_2CO_3<br>100.0; 0.72;<br>12	24	reflux	1.6; 3.0
2	26.7;<br>0.06;<br>1.0	57.0;<br>0.21;<br>3.5	THF (5)	$Pd(PPh_3)_4$<br>7.0; 0.006;<br>0.1	K_2CO_3<br>100.0; 0.72;<br>12	24	reflux	3.9; 7.0
3	26.7;<br>0.06;<br>1.0	57.0;<br>0.21;<br>3.5	THF (6)	$Pd(PPh_3)_4$<br>7.0; 0.006;<br>0.1	K_2CO_3<br>100.0; 0.72;<br>12	48	reflux.	3.4; 6.0
4	26.7;<br>0.06;<br>1.0	96.4;<br>0.36;<br>6.0	THF (5)	$Pd(dppf)Cl_2$<br>4.4; 0.006;<br>0.1	K_2CO_3<br>100.0; 0.72;<br>12	24	reflux	4.6; 7.6
5[b]	26.7;<br>0.06;<br>1.0	64.3;<br>0.24;<br>4.0<br>+<br>32.1;<br>0.12;<br>2.0	THF (5)	$Pd(dppf)Cl_2$<br>4.4; 0.006;<br>0.05<br>+<br>2.2; 0.003;<br>0.05	K_2CO_3<br>100.0; 0.72;<br>12	24	reflux	10.4;<br>40.0
6	26.7;<br>0.06;<br>1.0	96.4;<br>0.36;<br>6.0	DMF (6)	$Pd(dppf)Cl_2$<br>4.4; 0.006;<br>0.1	K_2CO_3<br>100.0; 0.72;<br>12	24	80°C	24.4;<br>**41.0**

[a] Isolated yield; [b] In this trial, new portion of **4** and Pd catalyst were added after 6 hours.

S2. Compounds characterization data

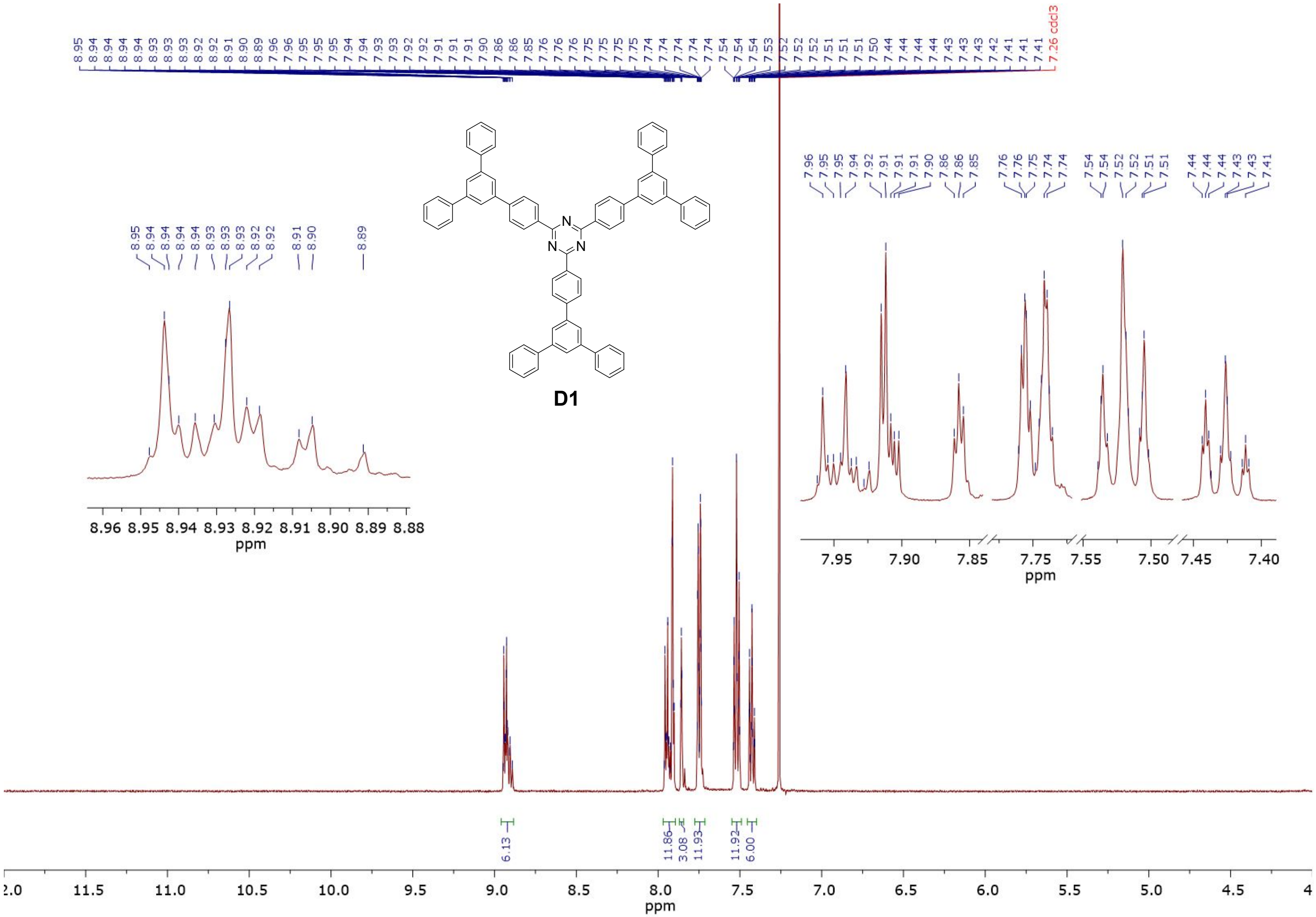

Figure S1. ^{1}H NMR spectrum (500 MHz, $CDCl_3$) of **D1**.

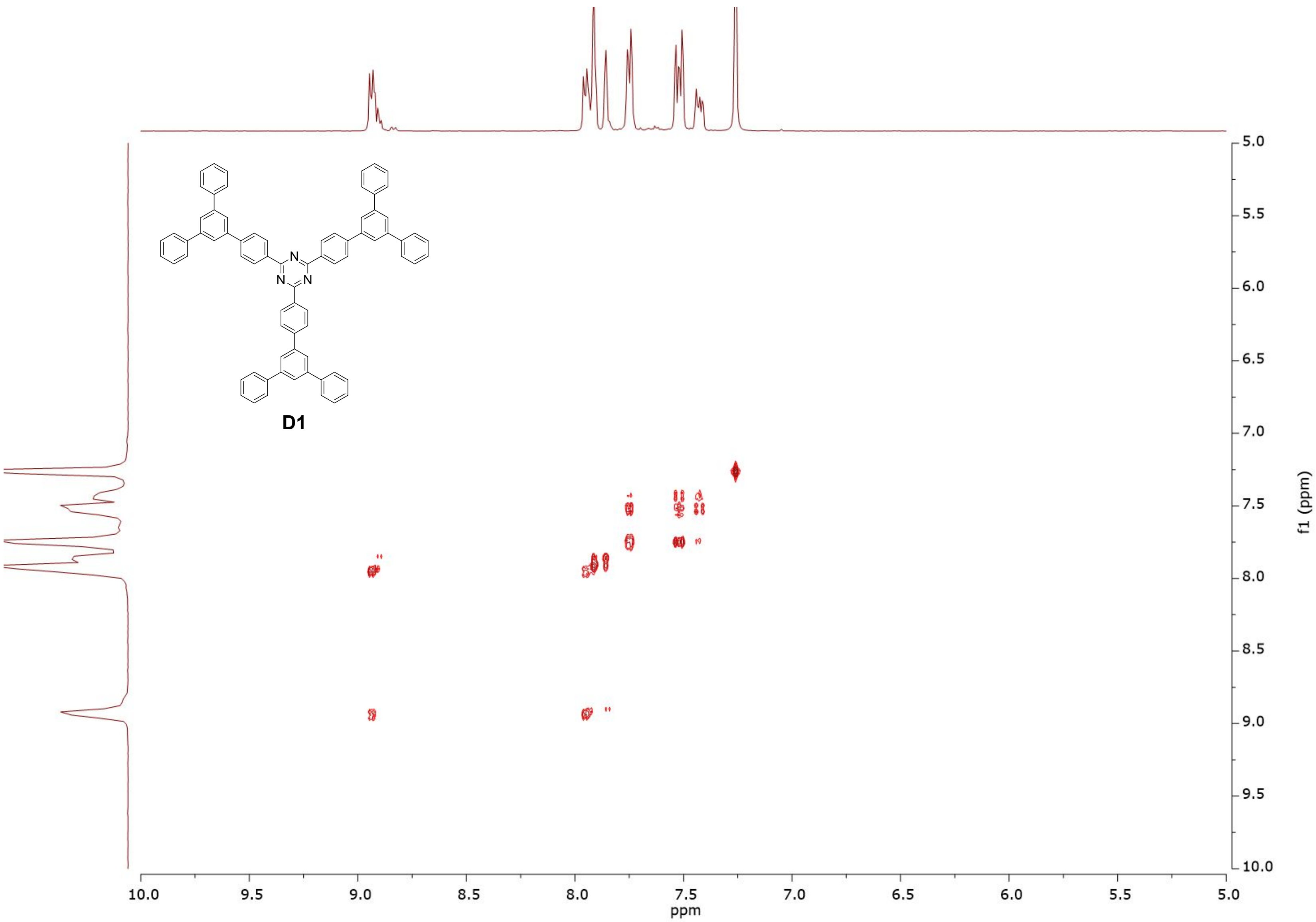

Figure S2. ^{1}H-^{1}H COSY NMR spectrum (500 MHz, $CDCl_3$) of **D1**.

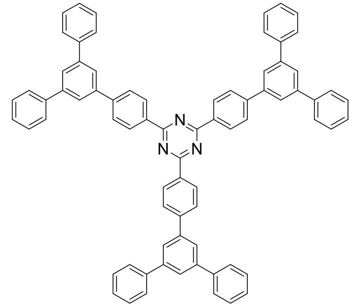

D1

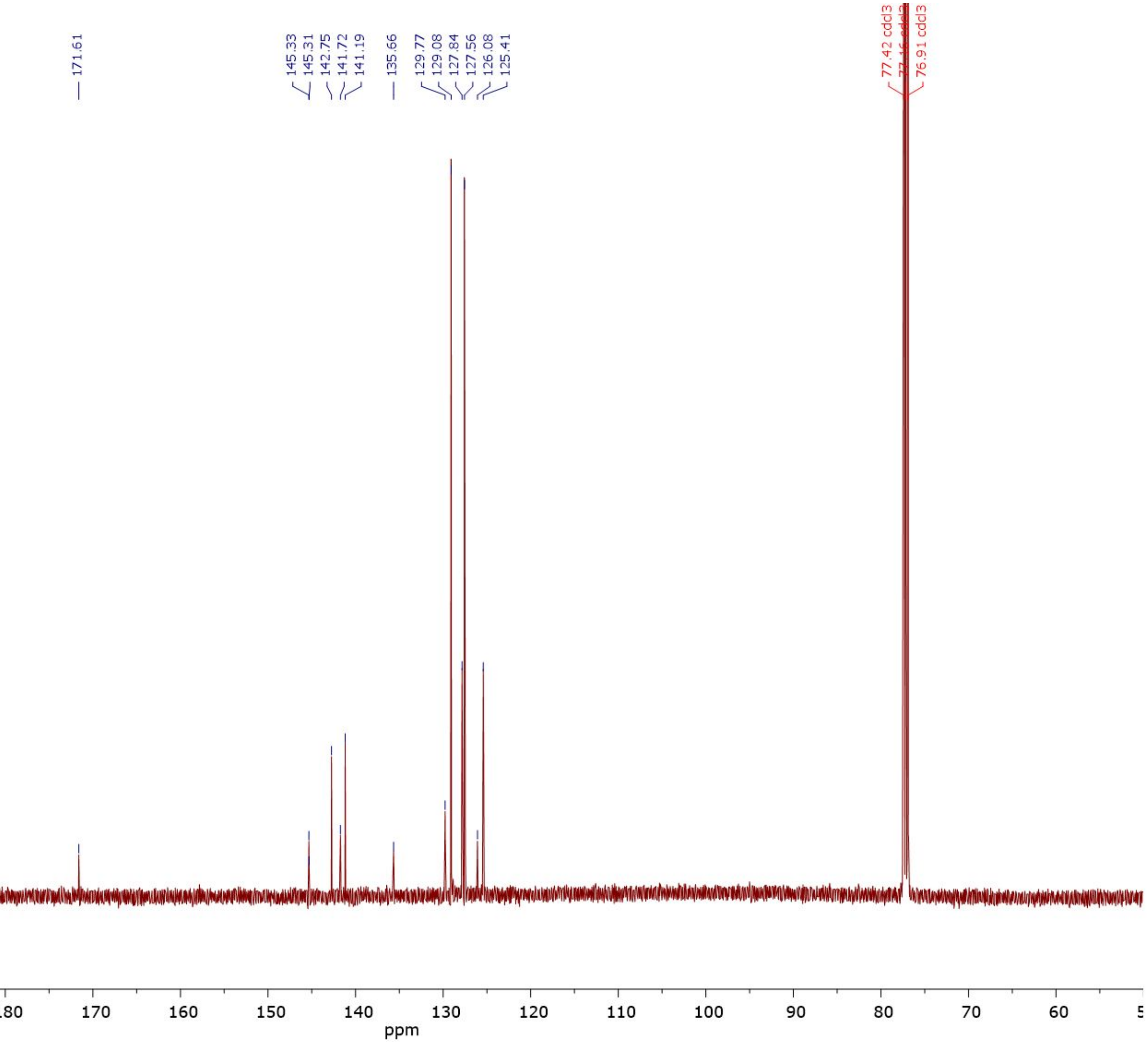

Figure S3. $^{13}C\{^{1}H\}$ NMR spectrum (125 MHz, $CDCl_3$) of **D1**.

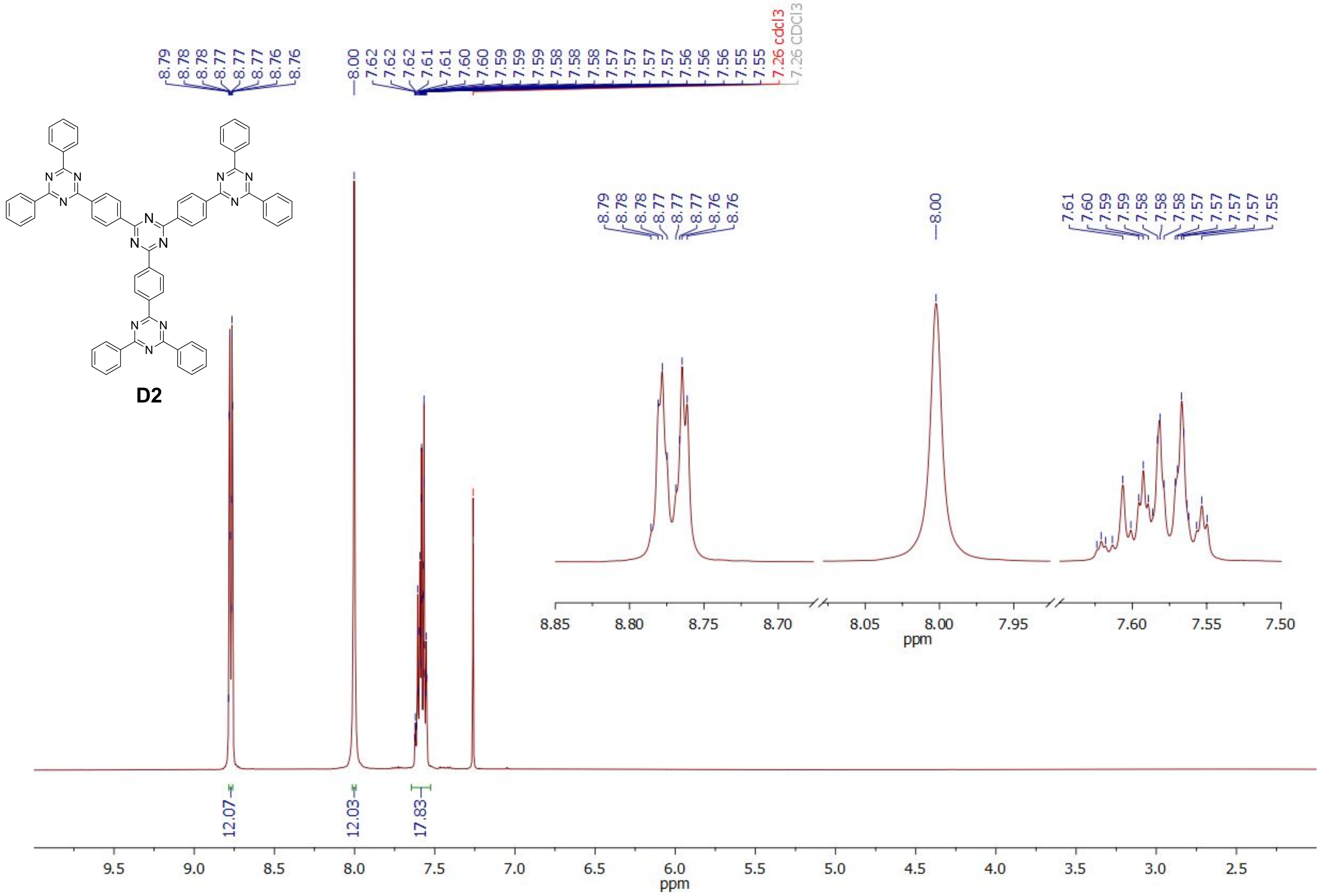

Figure S4. ^{1}H NMR spectrum (500 MHz, $CDCl_3$) of **D2**.

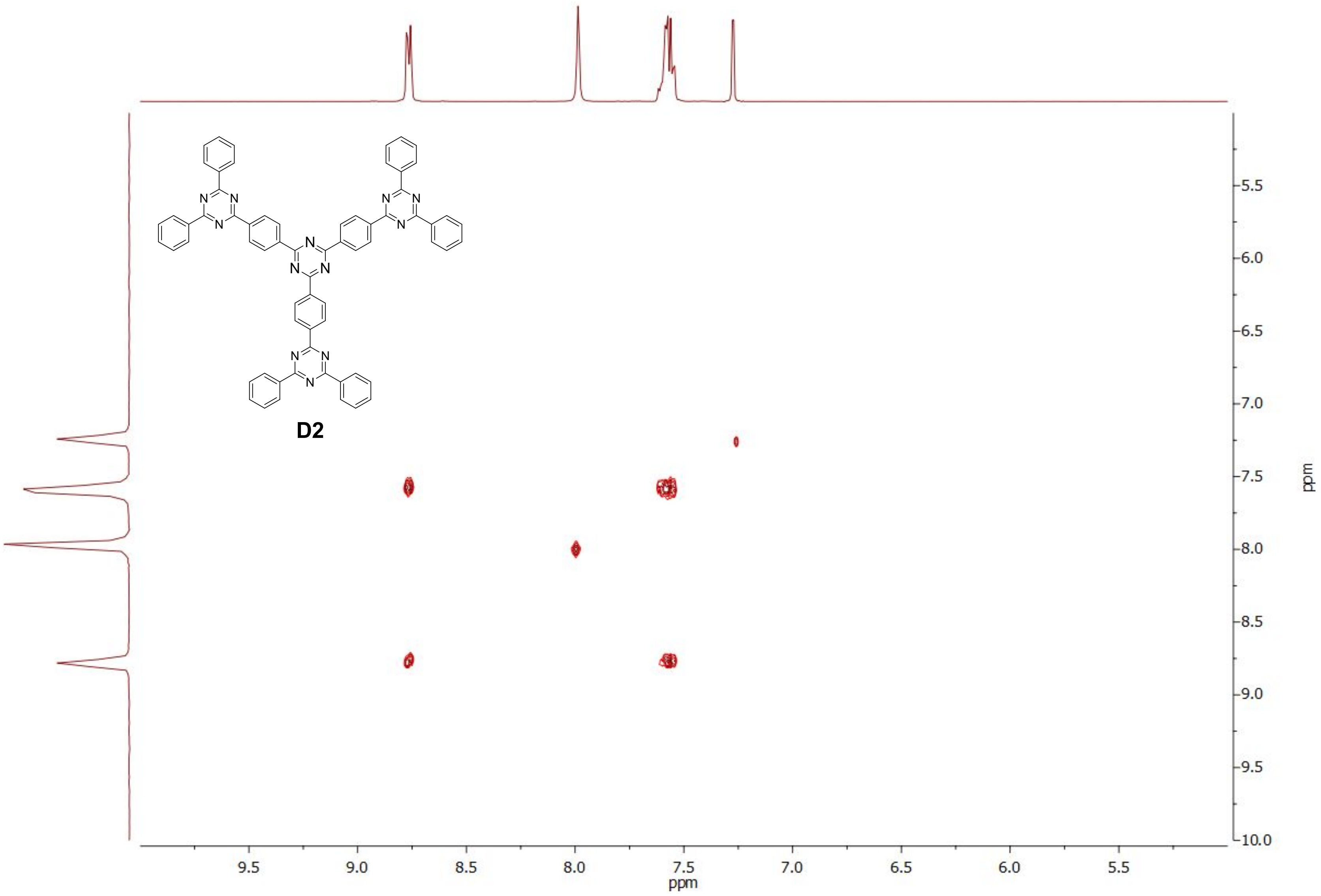

Figure S5. ^{1}H-^{1}H COSY NMR spectrum (500 MHz, $CDCl_3$) of **D2**.

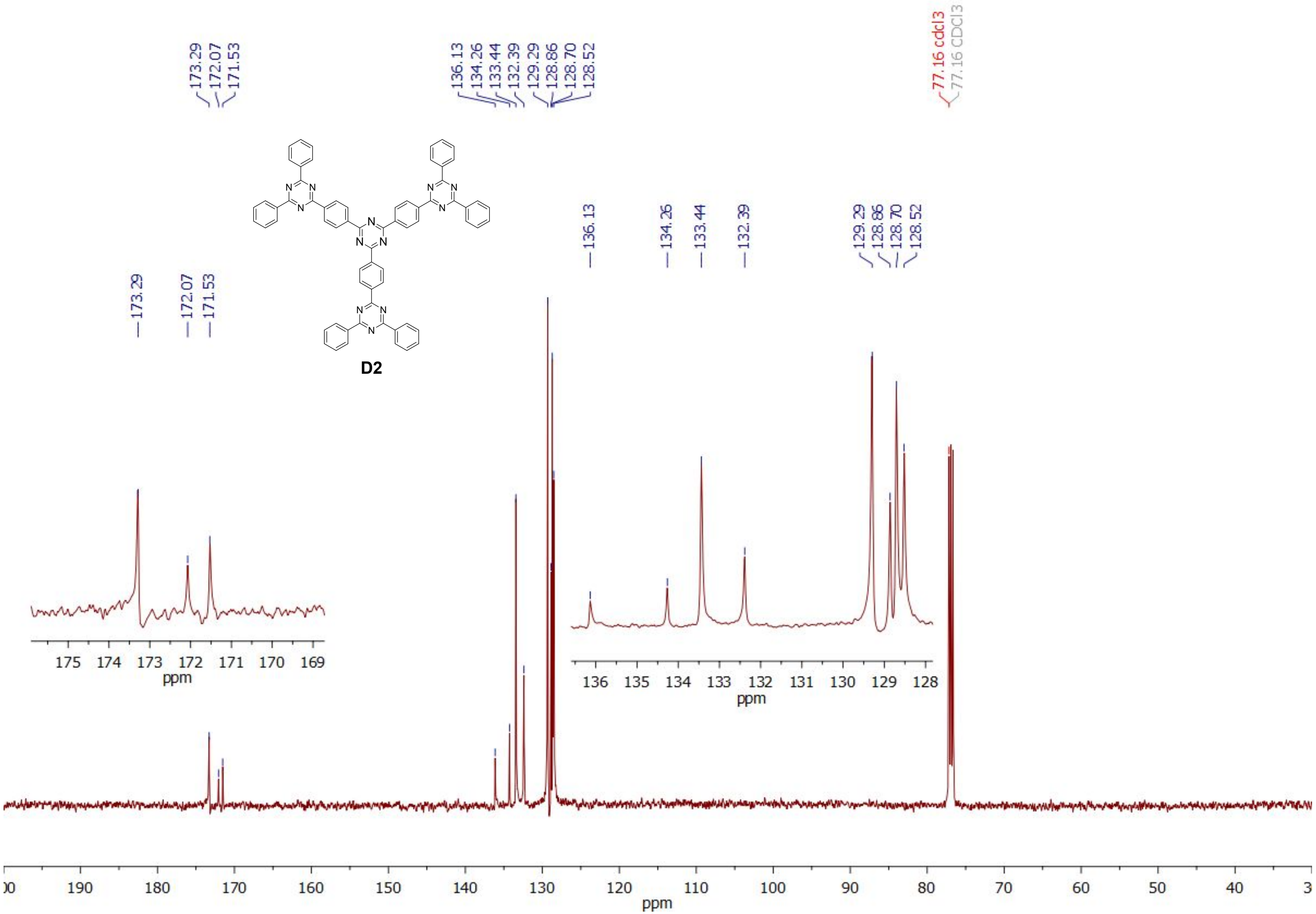

Figure S6. $^{13}C\{^{1}H\}$ NMR spectrum (125 MHz, $CDCl_3$) of **D2**.

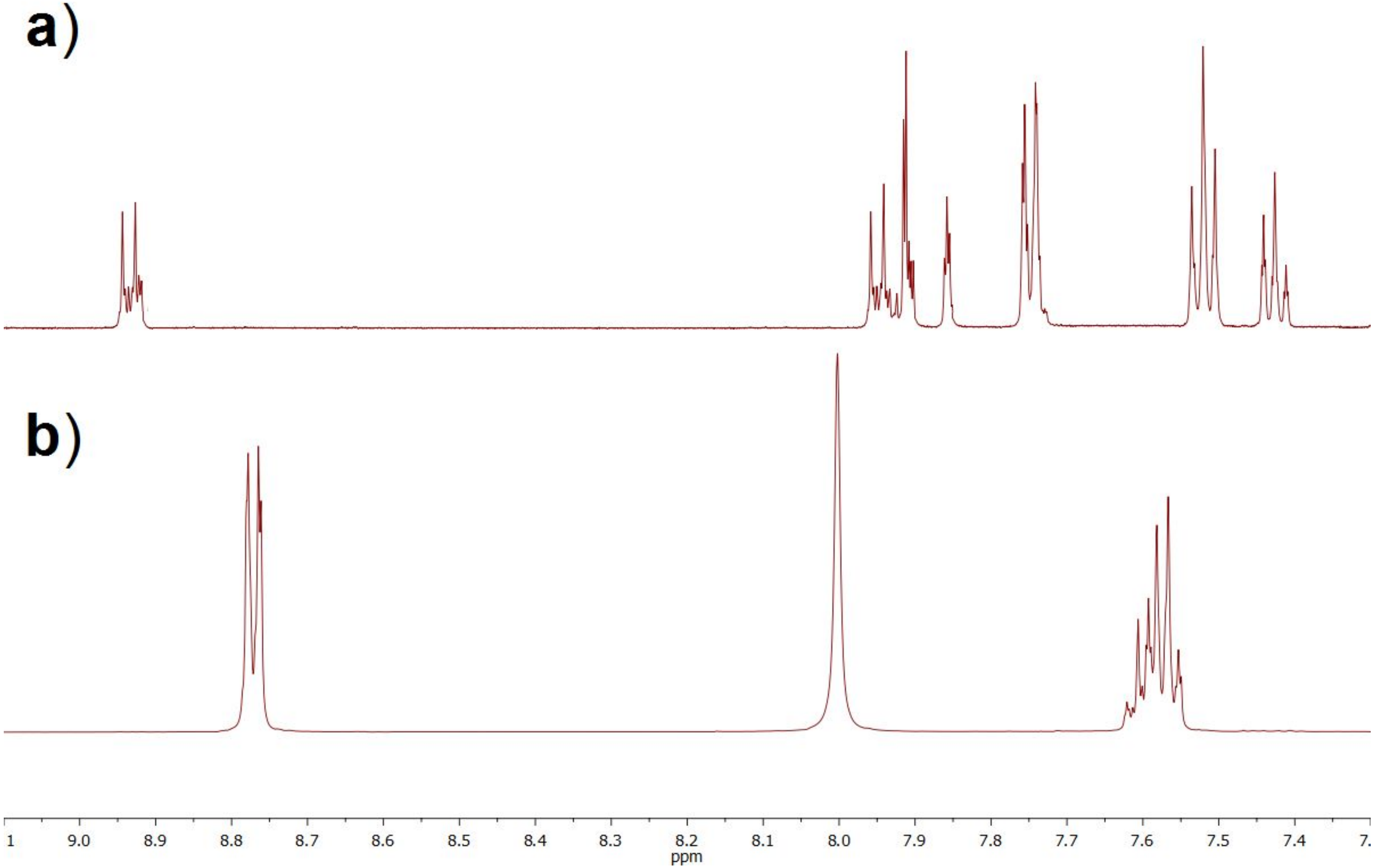

Figure S7. Comparison of ^{1}H NMR spectra (500 MHz, $CDCl_3$) of **D1** (**a**) and **D2** (**b**). 9.10-7.30 ppm insets are presented.

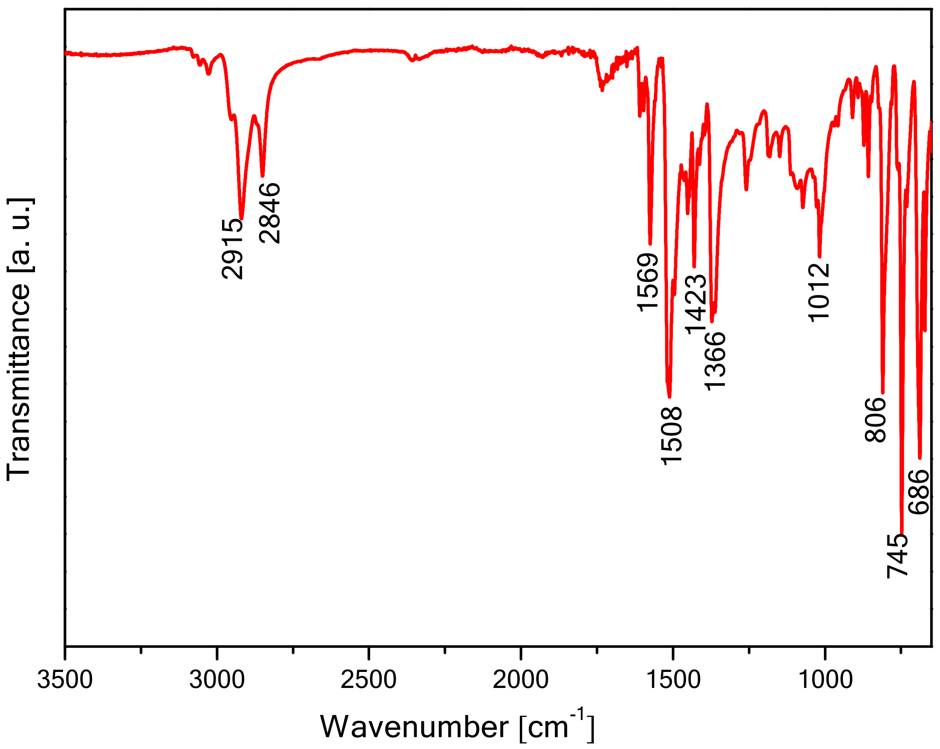

Figure S8. FT-IR spectrum of **D1**.

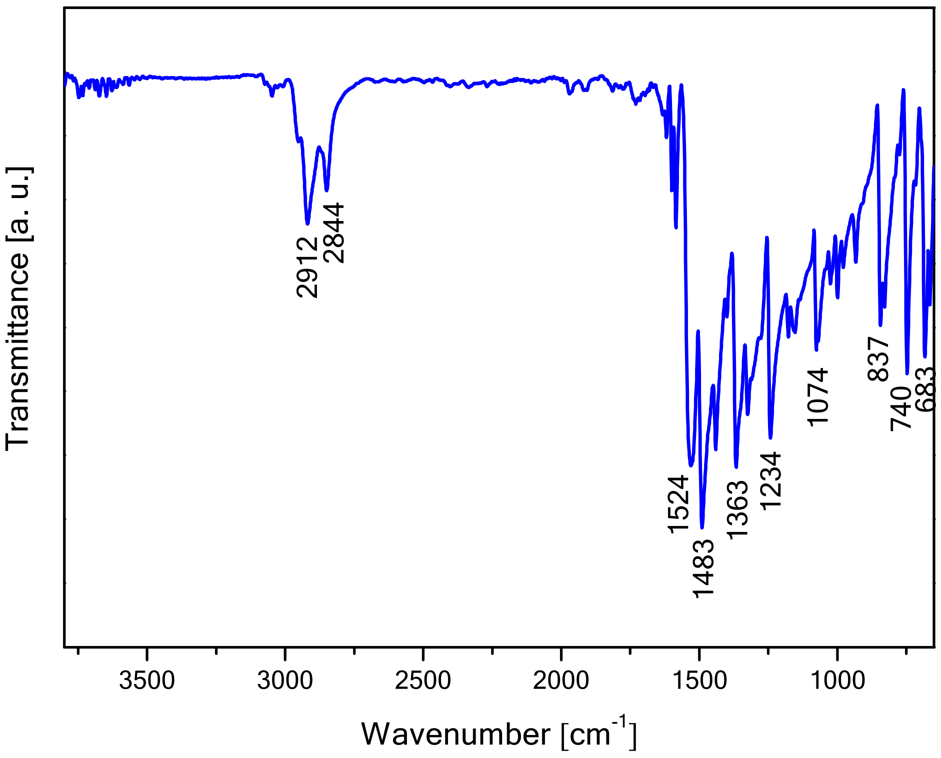

Figure S9. FT-IR spectrum of **D2**.

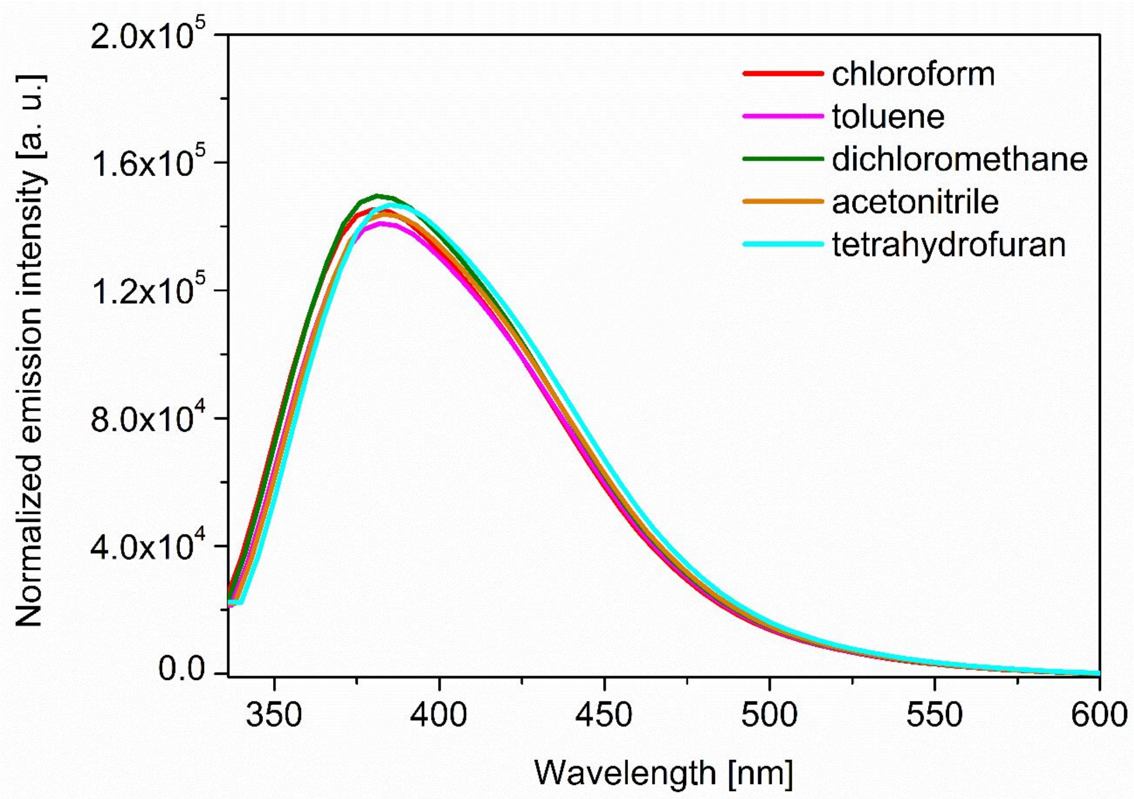

Figure S10. Emission spectra for **D1** measured in different solvents. Concentrations of the samples: $2 \cdot 10^{-5}$ M. λ_{ex} = 315 nm.

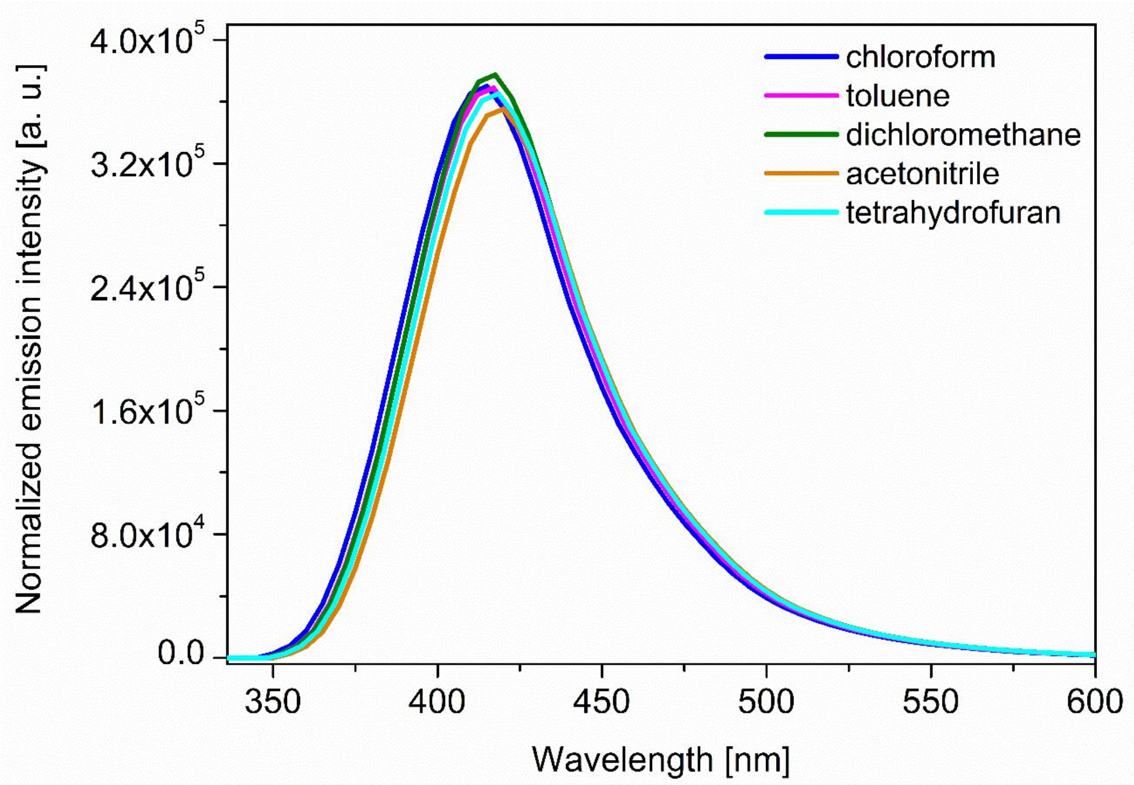

Figure S11. Emission spectra for **D2** measured in different solvents. Concentrations of the samples: $2 \cdot 10^{-5}$ M. λ_{ex} = 315 nm.

S3. Photocatalytic studies

S3.1 Photocatalytic synthesis of N-benzylidene benzylamines from benzylamines

Table S3. The results of the optimization experiments toward photocatalytic oxidation of benzylamine (**6**) using **D1** or **D2** as the photocatalyst.

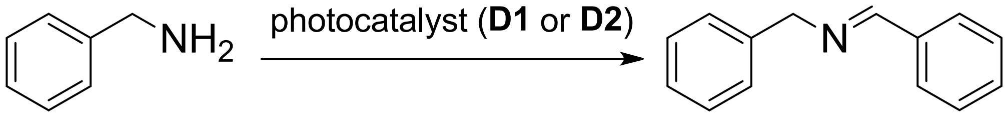

5 **6**

Entry	Photocatalyst[a] (type; mol%)	Reaction time (hours)	Conversion[b] (%)
1	**D1**; 0.5	1.5	52
2	**D2**; 0.5	1.5	78
3	**D2**; 0.5	2.0	83
4	**D2**; 1.0	3.0	87
5	**D2**; 1.5	3.0	94
6	**D2**; 2.0	3.0	>99[c]
7	-	3.0	0
8[d]	**D2**; 2.0	3.0	0
9[e]	**D2**; 2.0	3.0	47
10[f]	**D2**; 2.0	3.0	34
11[g]	**D2**; 2.0	3.0	20

[a] Reaction conditions: benzylamine (0.5 mmol), CH_2Cl_2 (5 mL), room temperature, air, UV-LED irradiation (365 nm). [b] Estimated with ^1H NMR. [c] Quantitative isolated yield (also in the reaction scale of 1.5 mmol). [d] Reaction without using UV-LED irradiation. [e] Reaction in the presence of benzoquinone (2.0 mol%). [f] Reaction in the presence of DMPO (2.0 mol%). [g] Reaction in the presence of DABCO (2.0 mol%).

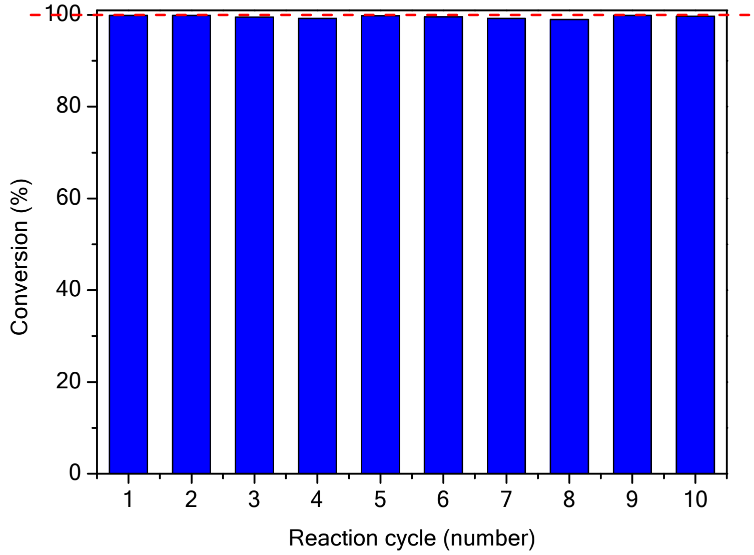

Figure S12. Data on the reusability studies with **D2** toward the synthesis of *N*-benzylidene benzylamine (**6**).

S3.2 Determination of conversion of benzylamine based on ^{1}H NMR spectra

The calculations were based on the literature[2] following reaction equation:

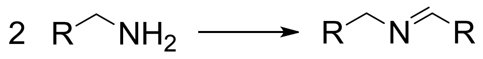

benz ***P***

Conversion (*C*) is defined as ratio of moles of benzylamine (*benz*) reacted (n_{benz}^{R}) to the initial number of moles of benzylamine (n_{benz}^{0}), *i.e.*, $C = \frac{n_{benz}^{R}}{n_{benz}^{0}}$. Two moles of benzylamine (*benz*) react to provide one mole of product (*P*; *N*-benzylidene benzylamine), *i.e.*, $n_{benz}^{R} = 2n_P$ and $n_{benz}^{0} = 2n_P + n_{benz}$.

Considering the above equations, *C* was estimated as follows:

$$C = \frac{2n_P}{2n_P + n_{benz}} = \frac{2}{2 + \frac{n_{benz}}{n_P}}$$

Number of moles of *benz* (n_{benz}) and *P* (n_P) were calculated as follows:

$$n_{benz} = \frac{\text{integral of } benz}{N_{benz}} \text{ and } n_P = \frac{\text{integral of } P}{N_P}$$

where N_{benz} and N_P stand for the number of protons represented by the given peak.

S3.3 ^{1}H NMR spectrum of the isolated N-benzylidene benzylamine (6) under optimized conditions

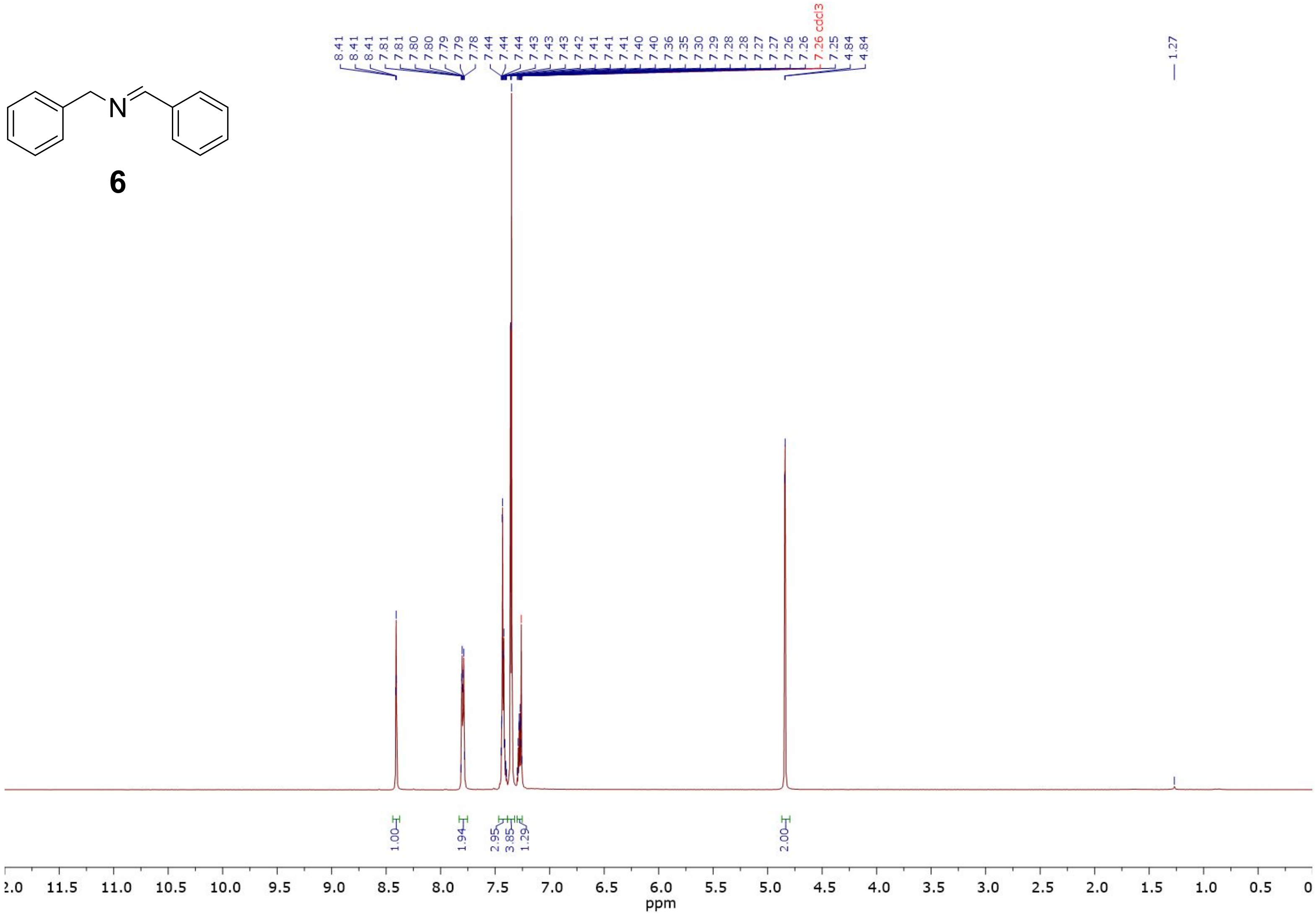

Figure S13. ^{1}H NMR spectrum (500 MHz, $CDCl_3$) of isolated *N*-benzylidene benzylamine (**6**) from the photocatalytic reaction.

S34.4 Determination of HOMO-LUMO band gaps for D1 and D2

Tauc plot toward HOMO-LUMO band gap energy determination[3] for **D1** and **D2** are presented in Figure S14 and Figure S15.

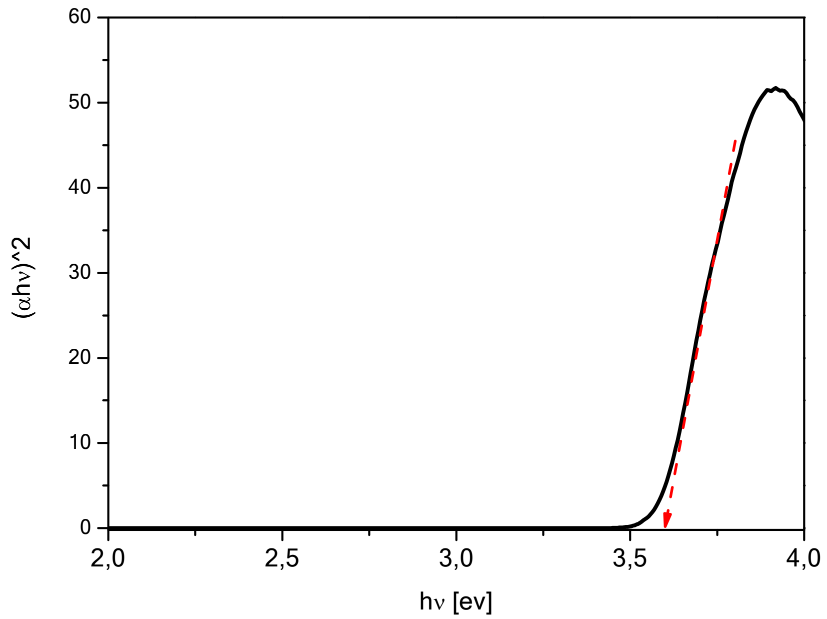

Figure S14. Tauc plot for **D1**. The linear part of the plot is extrapolated to the *x*-axis.

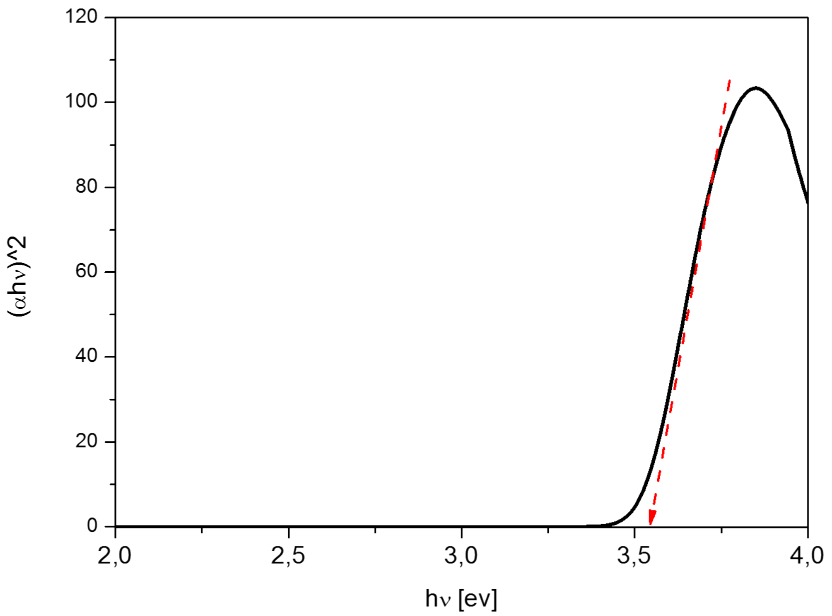

Figure S15. Tauc plot for **D2**. The linear part of the plot is extrapolated to the *x*-axis.

S3.5 Plausible literature reaction mechanism

The plausible reaction mechanism of the studied photocatalytic reaction[1] is presented in Figure S16.

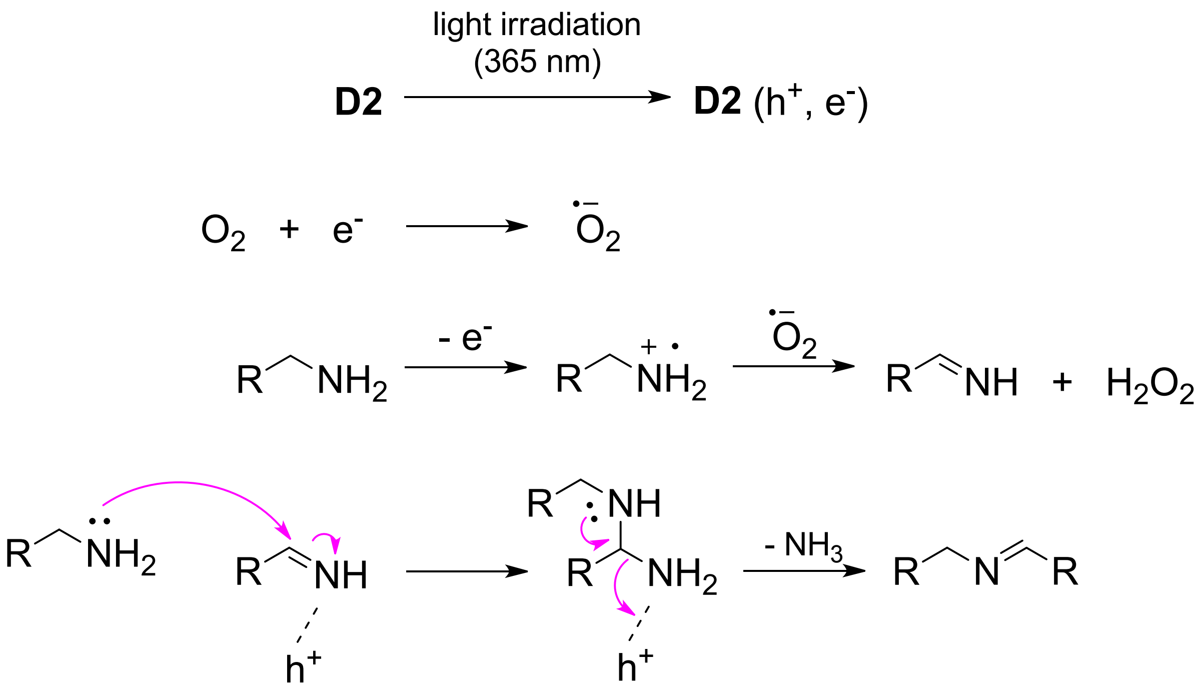

Figure S16. Plausible literature reaction mechanism for the studied photocatalytic coupling (**D2** was included as the representative photocatalyst).

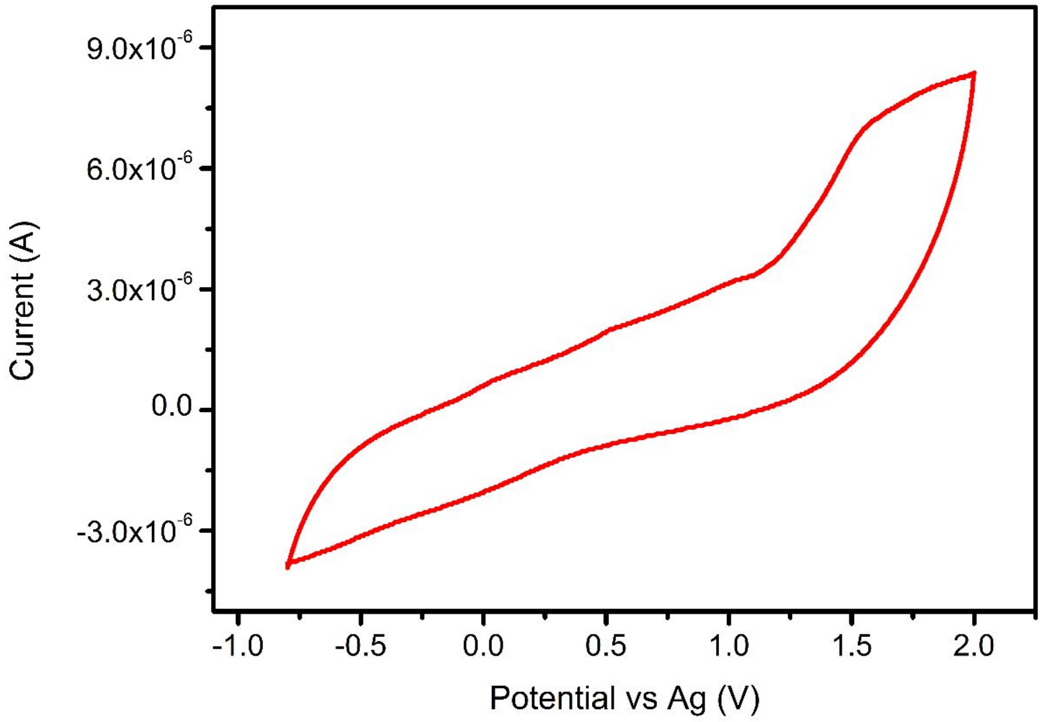

Figure S17. Cyclic voltammogram for **D1**.

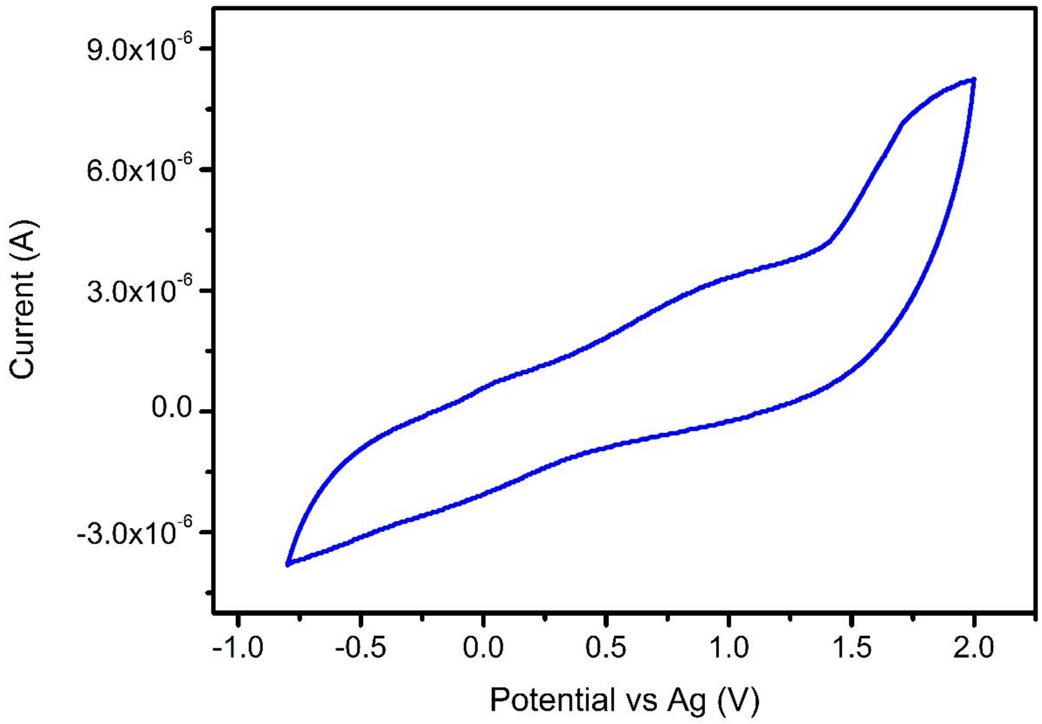

Figure S18. Cyclic voltammogram for **D2**.

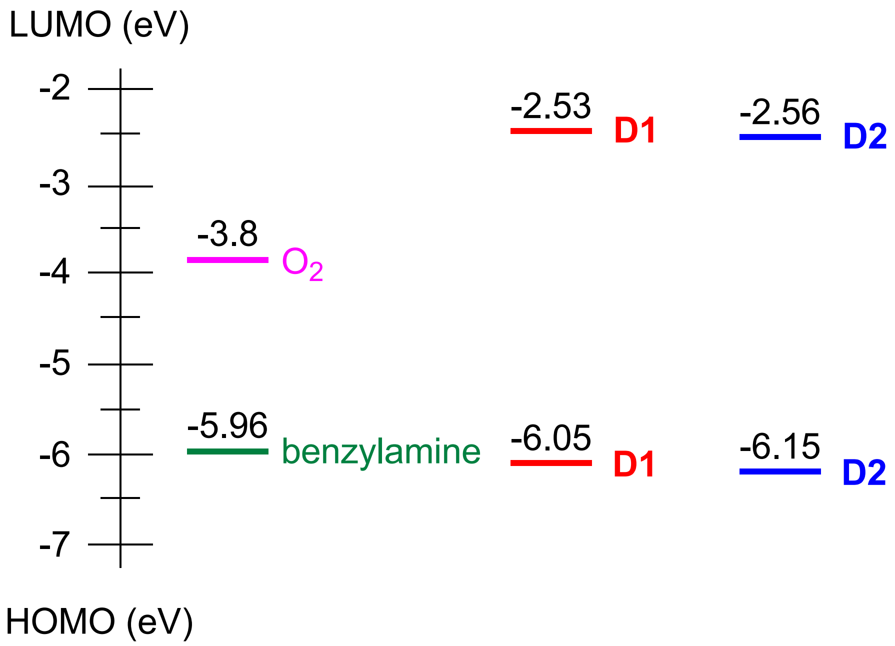

Figure S19. Schematic representation of the HOMO/LUMO energy levels of **D1** and **D2**. HOMO (eV) and LUMO (eV) values for **D1** and **D2** were calculated as follows[2] : HOMO = $-[E_{\mathrm{ox}}^{\mathrm{onset}} + 4.8]$ eV, where $E_{\mathrm{ox}}^{\mathrm{onset}}$ was calculated from the cyclic voltammetry measurements, LUMO = $-[\mathrm{HOMO} + E_{\mathrm{eg}}^{\mathrm{opt}}]$, where $E_{\mathrm{eg}}^{\mathrm{opt}}$ was calculated the absorption onset. Please note that these band gaps and the respective values calculated from the Tauc plot method (Figures S14, S15) were highly consistent.

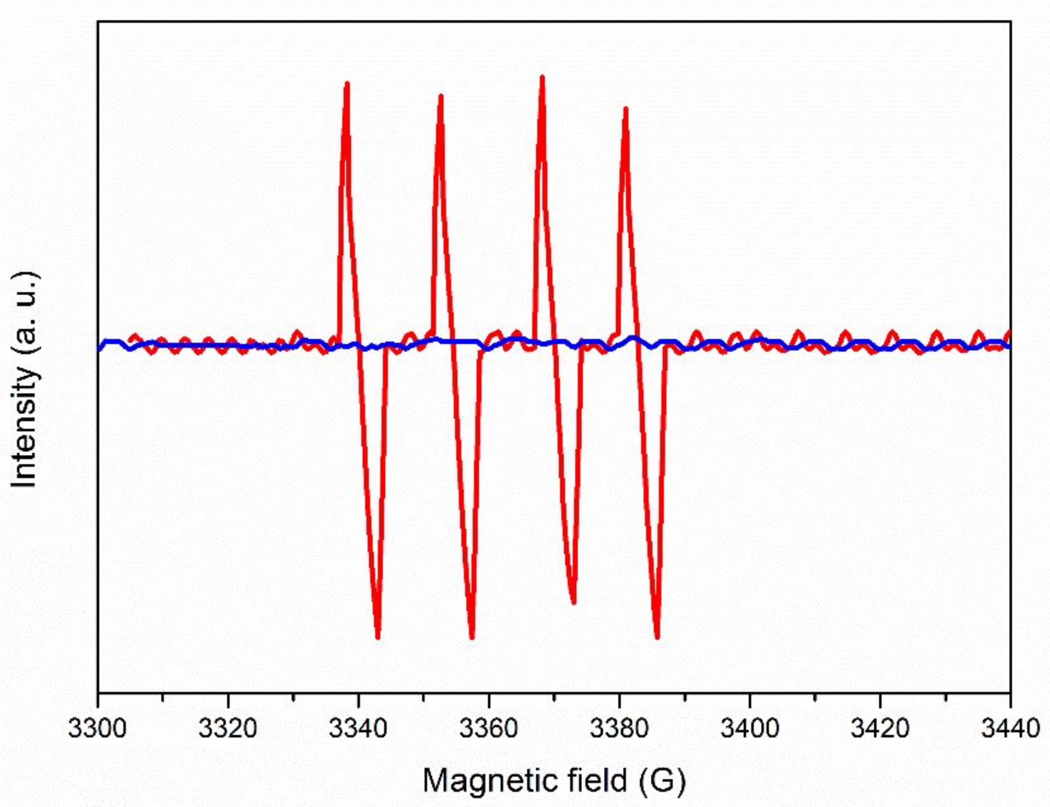

Figure S20. EPR spectra of DMPO-$O_2^{\bullet -}$. The spectrum was measured at room temperature with irradiation (red line) or without irradiation (blue line).

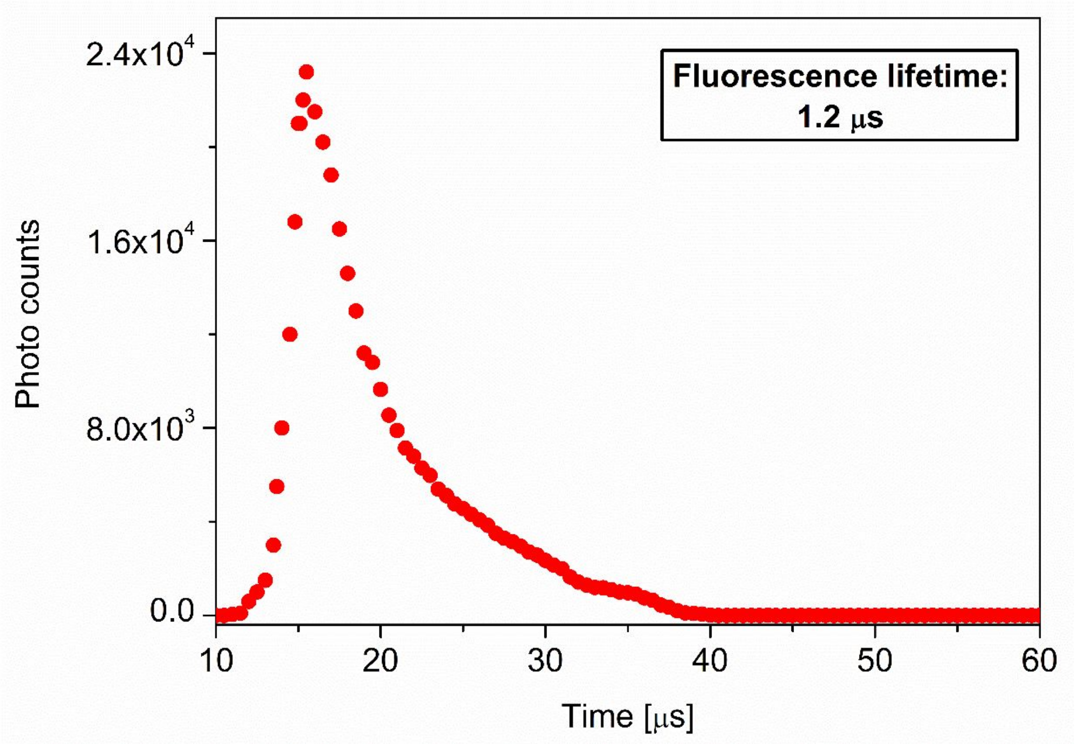

Figure S21. Fluorescence lifetime curve for **D1.** The fluorescence lifetime parameter is presented in the figure.

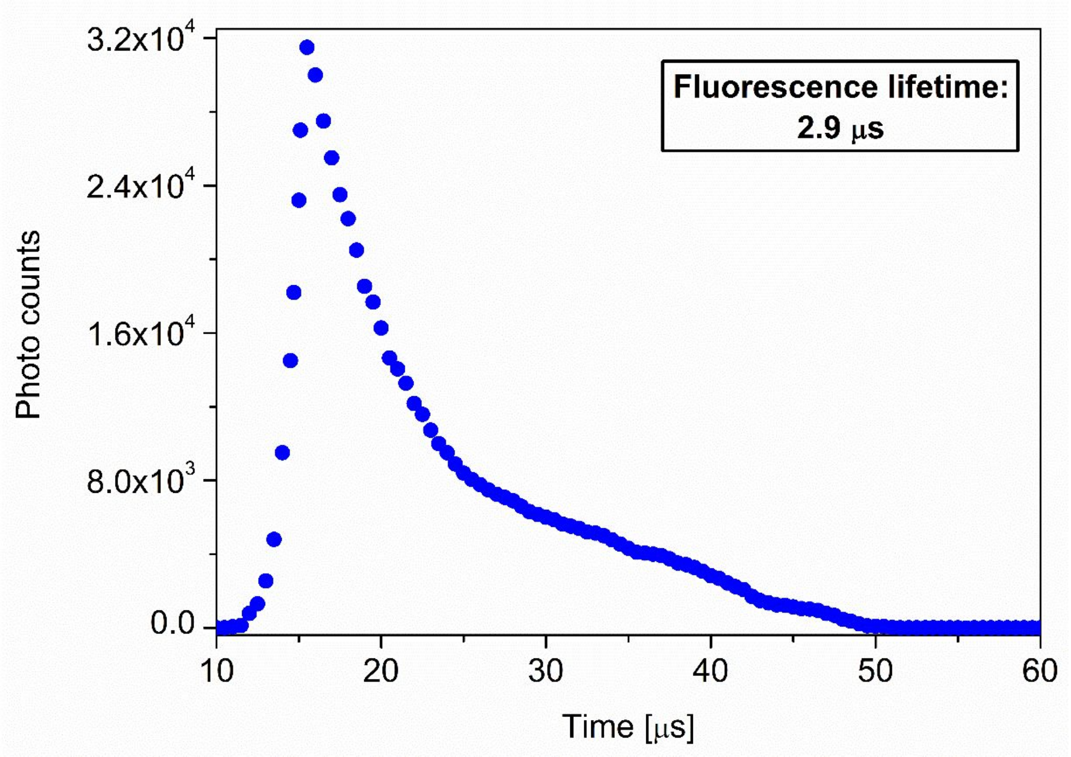

Figure S22. Fluorescence lifetime curve for **D2.** The fluorescence lifetime parameter is presented in the figure.

S4. TGA curves for D1 and D2

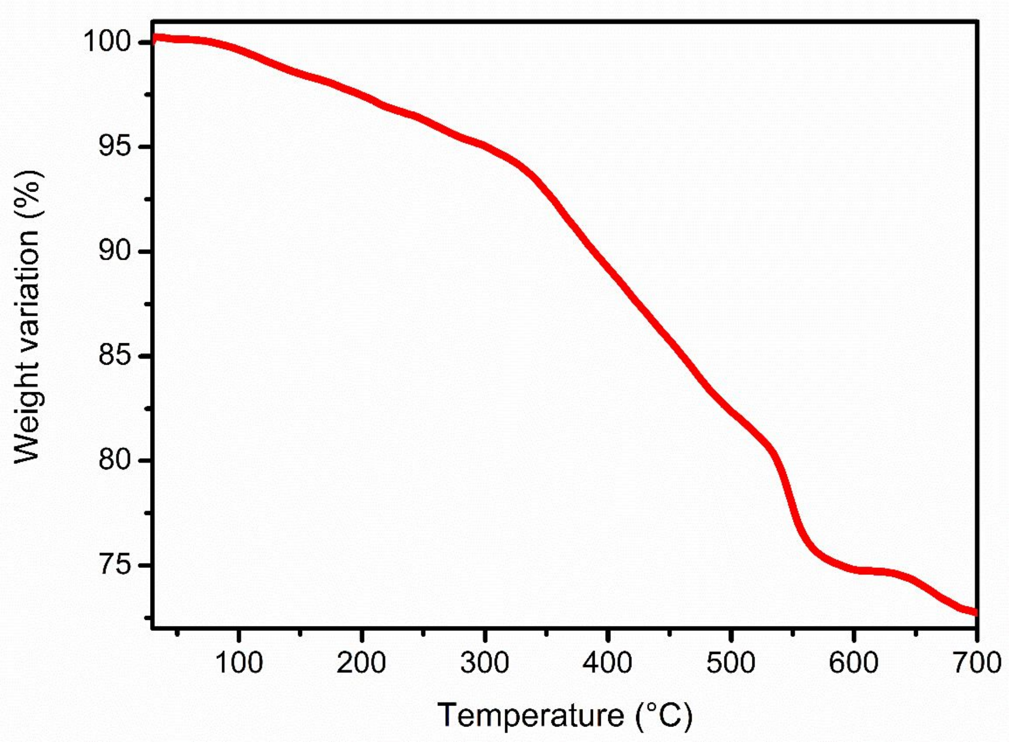

Figure S23. TGA curve (in argon) for **D1**.

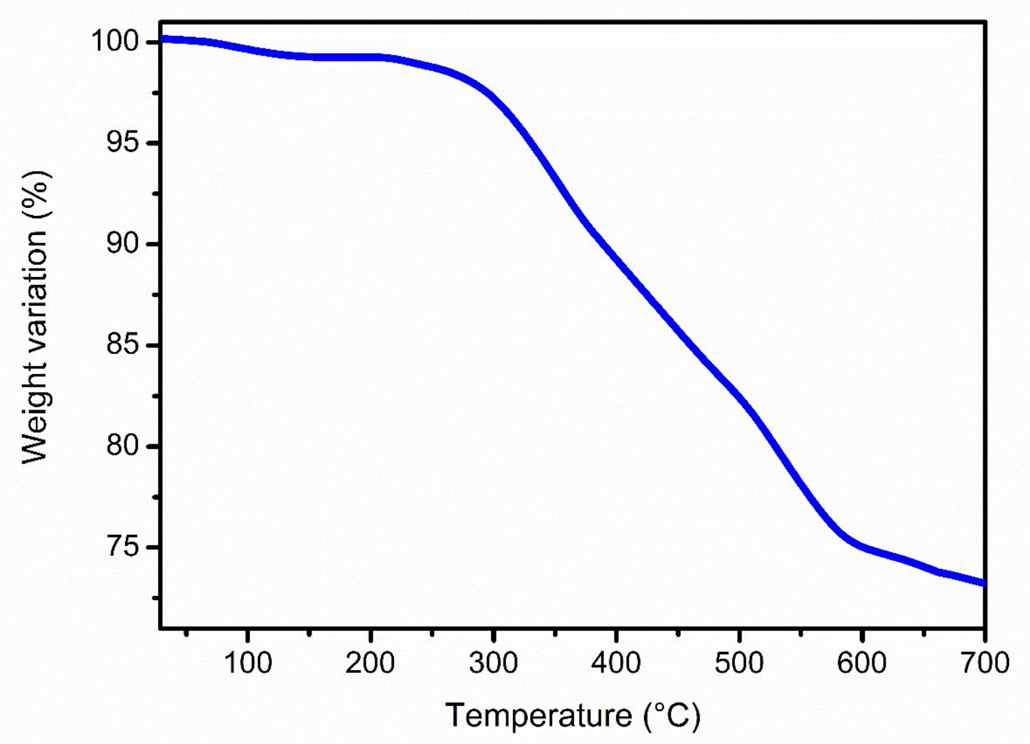

Figure S24. TGA curve (in argon) for **D2**.

S5. UV-Vis spectra

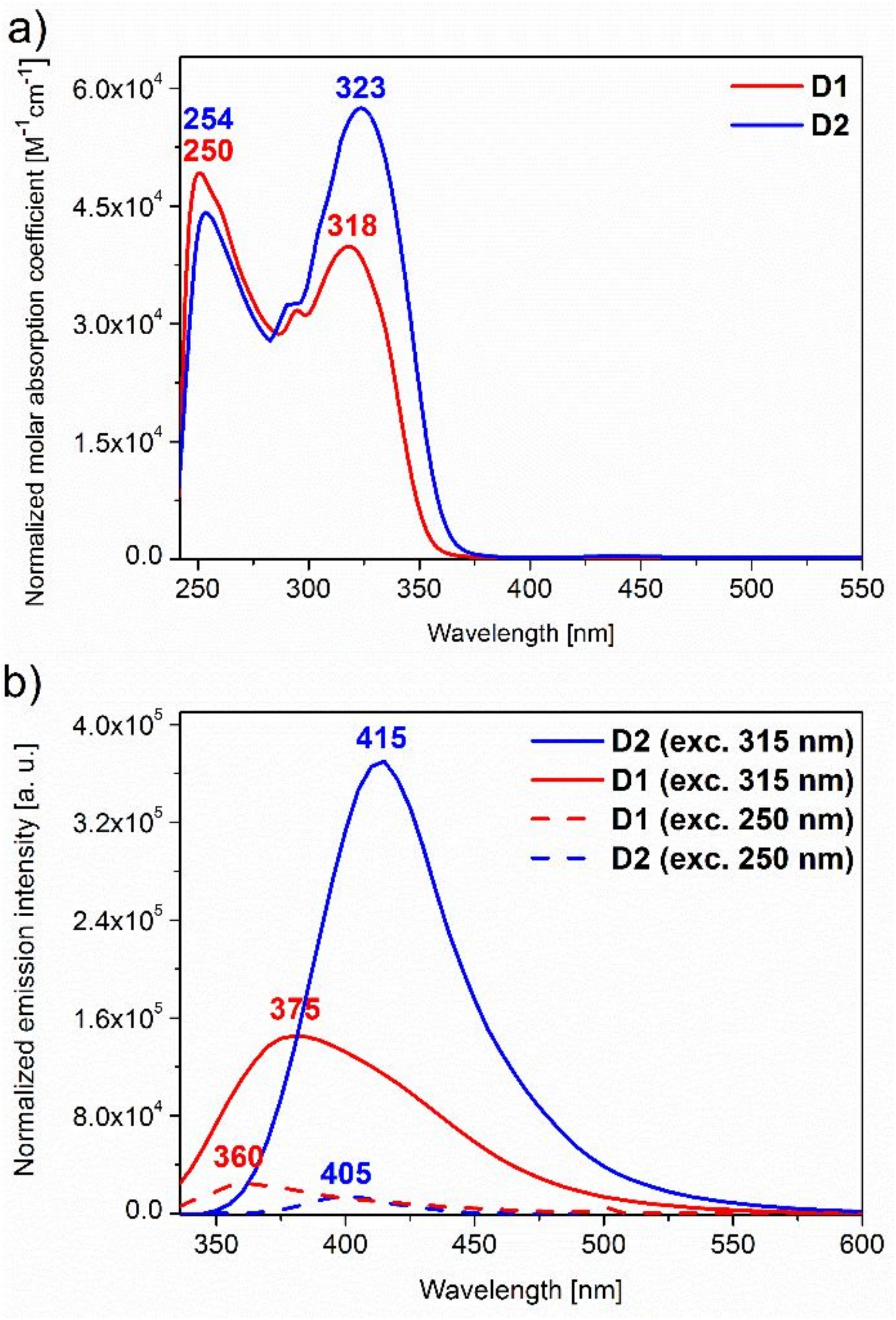

Figure S25. (**a**) UV-Vis and (**b**) emission spectra for **D1** and **D2**. Concentrations of the samples: $2{\cdot}10^{-5}$ M ($CHCl_3$).

S6. OLED application trials with D1 and D2

The construction of the devices was based on the previously reported protocol[4] with slight modifications. Clean indium tin oxide (ITO) was died in 130°C for 20 min and then treated with UV-Ozone for 7 minutes. Then, a layer of poly(3,4-ethylnenedioxythiophene):poly(styrenesulfonate) (PEDOT:PSS) was prepared by spin coating onto the ITO at 700 rpm for 10 seconds and 3500 rpm for 90 seconds. It was then heated at 130°C for 40 minutes. In the meantime, an excess of a $CHCl_3$ solution of **D1** or **D2** (7 mg·mL^{-1}) was added to the stock solution of 9,9'-(1,3-phenylene)-bis-9H-carbazole (MCP) in $CHCl_3$ to the final concentration of 25 wt%. This solution was spincoated on the ITO/PEDOT:PSS later and the as-obtained samples were placed into a deposition chamber. Tri[(3-pyridyl)-phen-3-yl]benzene (TmPyPb), LiF and Al were deposited thermally (2·10^5 mbar). The emission area of the devices equaled to 0.15-0.16 cm^2. OLED device comprising **D1** was labeled as 'OLED-D1', whilst the respective device consisting of **D2** was labelled as 'OLED-D2'. It was found that OLED-D1 emission maximum was placed at 380 nm, whilst for OLED-D2 this value equaled to 410 nm.

Luminescence-voltage (L-V) and current density-voltage (J-V) curves for device OLED-D1 and OLED-D2 are presented in Figure S26.

Summary of selected working parameters of the constructed OLEDs together with the comparison with the other literature examples of OLEDs comprising aromatic dendrimers are presented in Table S4.

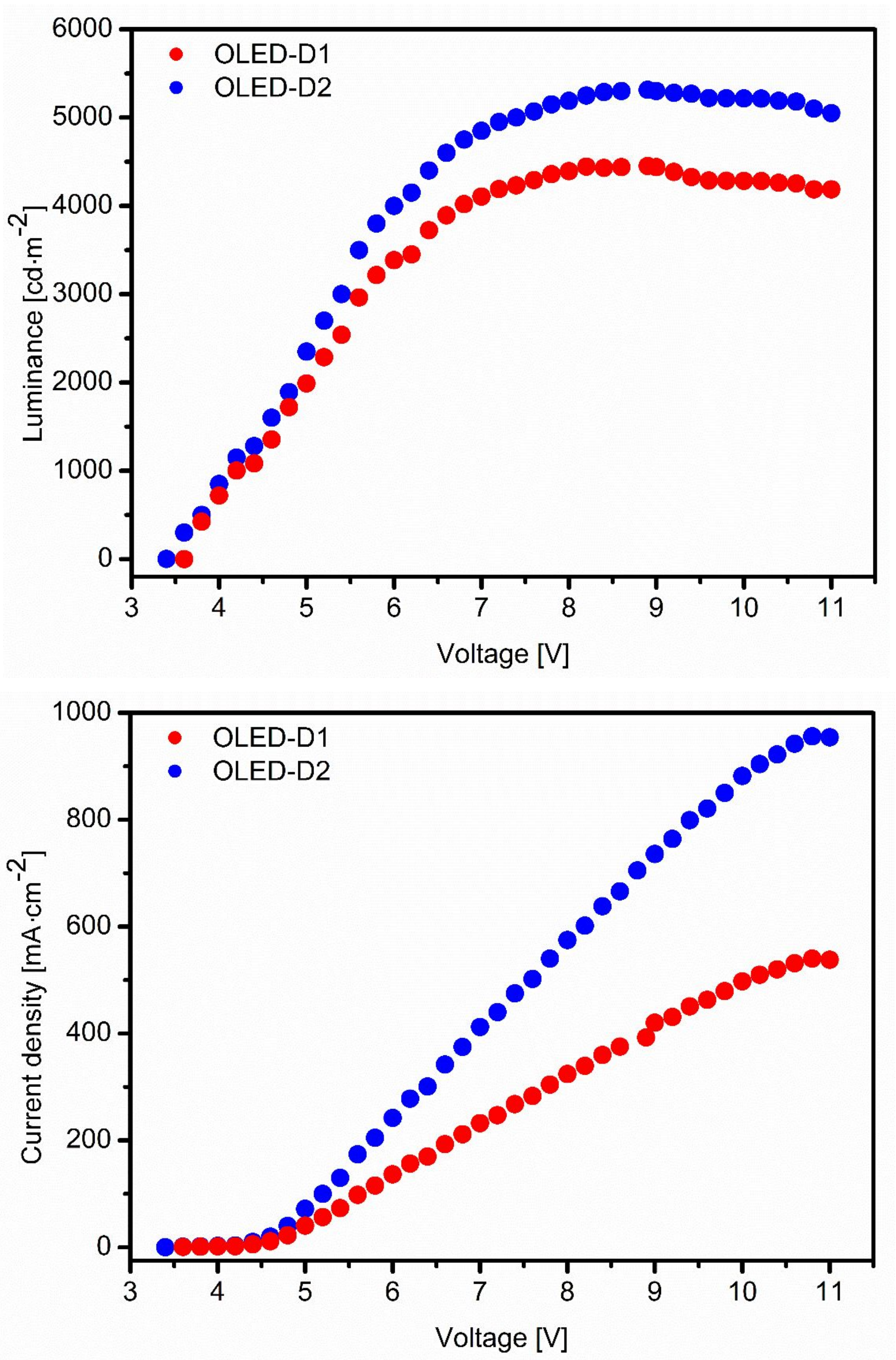

Figure S26. Luminescence-voltage (L-V) (top) and current density-voltage (J-V) (bottom) profiles for device OLED-D1 (red) and OLED-D2 (blue).

Table S4. Summary of selected working parameters of the constructed OLEDs together with the comparison with the other literature examples of OLEDs comprising aromatic dendrimers.

**Device**	**Emmision maximum [nm]**	**Maximum external quantum efficiency [%]**	**Turn-on-voltage [V]**	**Maximum luminance [cd·m^{-2}]**	**Ref.**
**OLED-D1**	380	0.85	3.6	4302	**This work**
**OLED-D2**	410	2.82	3.4	5585	**This work**

Literature examples of other OLED devices comprising an aromatic dendrimer

**Compound**	**Emmision maximum [nm]**	**Maximum external quantum efficiency [%]**	**Turn-on-voltage [V]**	**Maximum luminance [cd·m^{-2}]**	**Ref.**
Pyrene-containing 1,3,5-triazine based dendrimers	451-460	0.97	3.5	1273-4306	[4]
Phenoxazine-containing 1,3,5-triazine based dendrimers	520-580	Not given	Not given	Not given	[5]
Carbazole-containing 1,3,5-triazine based dendrimers	546-552	1.2-6.5	2.4-2.7	7942-25085	[6]
Carbazole-containing 1,3,5-triazine based dendrimers	500-550	2.8-20.6	3.0-3.2	1441-8963	[7]
Fluorene-containing 1,3,5-triazine based dendrimers	436-460	0.83-0.99	3.0-3.5	1610-3093	[8]

S7. ESI-HRMS spectra of D1 and D2

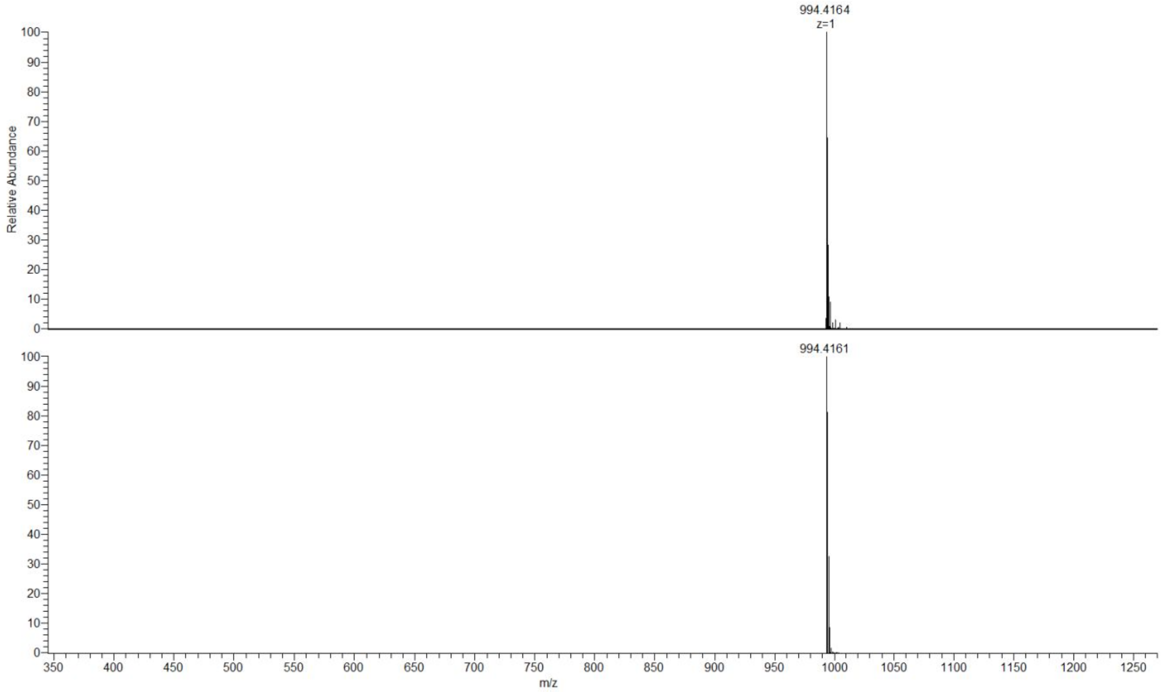

Figure S27. HRMS (ESI-TOF) of **D1** (top – measured, bottom – calculated).

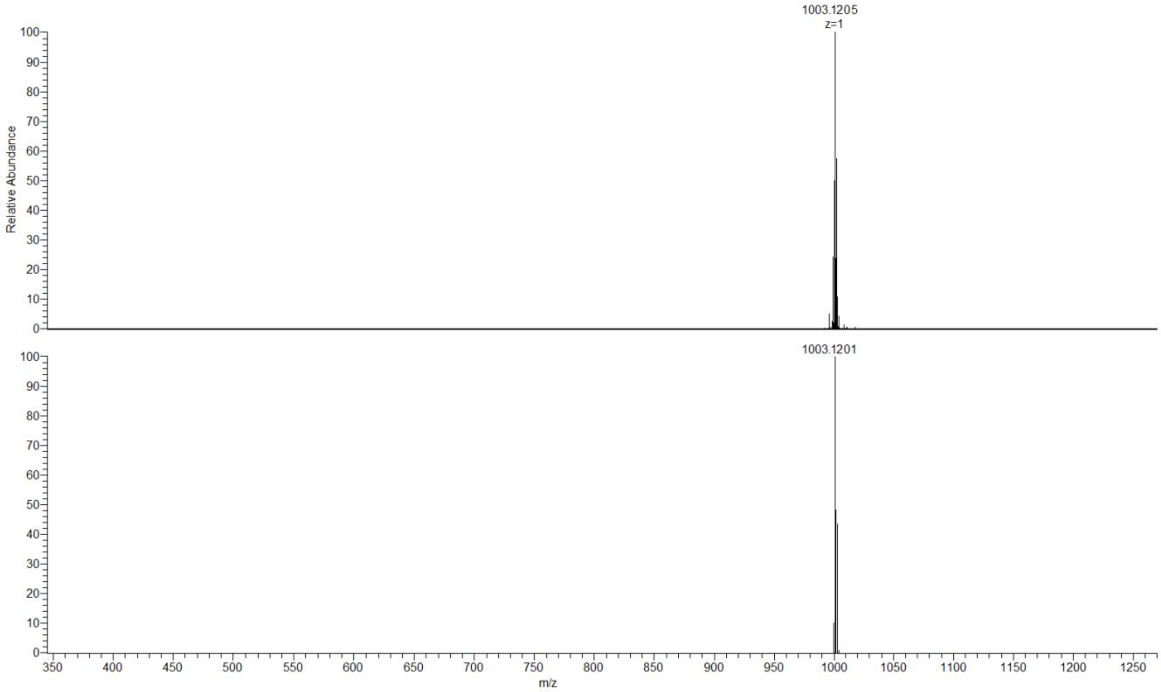

Figure S28. HRMS (ESI-TOF) of **D2** (top – measured, bottom – calculated).

S8. References

[1] Su, F.; Mathew, S. C.; Mohlmann, L.; Antonietti, M.; Wang, X.; Blechert, S. Aerobic Oxidative Coupling of Amines by Carbon Nitride Photocatalysis with Visible Light. *Angew. Chem. Int. Ed*. **2011**, *50*, 657–660.

[2] Bohra, H.; Li, P.; Yang, C.; Zhao, Y.; Wang, M. “Greener” and modular synthesis of triazine-based conjugated porous polymers via direct arylation polymerization: structure–function relationship and photocatalytic application. *Polym. Chem*. **2018**, *9*, 1972–1982.

[3] Makuła, P.; Pacia, M.; Macyk, W. How To Correctly Determine the Band Gap Energy of Modified Semiconductor Photocatalysts Based on UV–Vis Spectra. *J. Phys. Chem. Lett.* **2018**, *9*, 6814–6817.

[4] Li, Y. F.; Xie, X.; Gong, H. J.; Liu, M. L.; Chen, R. F.; Gao, D. Q.; Huang, W. Two bipolar blue-emitting fluorescent materials based on 1,3,5-trizine and peripheral pyrene for light-emitting diodes. *Dyes Pigm*. **2017**, *145*, 43-53.

[5] Tanaka, H.; Shizu, K.; Nakanotani, H.; Adachi, C. Twisted intramo-lecular charge transfer state for long-wavelengh thermally acti-vated delayed fluorescence. *Chem. Mater*. **2013**, *25*, 3766-3771.

[6] Sun, K.; Sun, Y.; Huang, T.; Luo, J.; Jiang, W.; Sun, Y. Design strategy of yellow thermally activated delayed fluorescent dendrimers and their highly efficient non-doped solution-processed OLEDs with low driving voltage. *Org. Electron*. **2017**, *42*, 123-130.

[7] Ma, Z.; Wan, Y.; Dong, W.; Si, Z.; Duan, Q.; Shao, S. Alkoxy encapsula-tion of carbazole-based thermally activated delayed fluorescent dendrimers for highly efficient solution-processed organic light emitting diodes. *Chin. Chem. Lett*. **2021**, *32*, 703-707.

[8] Ren, S.; Zeng, D.; Zhong, H.; Wang, Y.; Qian, S.; Fang, Q. Star-Shaped Donor-π-Acceptor Conjugated Oligomers with 1,3,5-Triazine Cores: Convergent Synthesis and Multifunctional Properties. *J. Phys. Chem. B* **2010**, *114*, 10374–10383.
